# BdLT-Seq as a barcode decay-based method to unravel lineage-linked transcriptome plasticity

**DOI:** 10.1038/s41467-023-36744-1

**Published:** 2023-02-25

**Authors:** Yelyzaveta Shlyakhtina, Bianca Bloechl, Maximiliano M. Portal

**Affiliations:** Cell Plasticity & Epigenetics Lab, Cancer Research UK – Manchester Institute, The University of Manchester, SK10 4TG Manchester, UK

**Keywords:** Epigenetics, Transcriptomics, RNA sequencing, Evolutionary theory

## Abstract

Cell plasticity is a core biological process underlying a myriad of molecular and cellular events taking place throughout organismal development and evolution. It has been postulated that cellular systems thrive to balance the organization of meta-stable states underlying this phenomenon, thereby maintaining a degree of populational homeostasis compatible with an ever-changing environment and, thus, life. Notably, albeit circumstantial evidence has been gathered in favour of the latter conceptual framework, a direct observation of meta-state dynamics and the biological consequences of such a process in generating non-genetic clonal diversity and divergent phenotypic output remains largely unexplored. To fill this void, here we develop a lineage-tracing technology termed Barcode decay Lineage Tracing-Seq. BdLT-Seq is based on episome-encoded molecular identifiers that, supported by the dynamic decay of the tracing information upon cell division, ascribe directionality to a cell lineage tree whilst directly coupling non-genetic molecular features to phenotypes in comparable genomic landscapes. We show that cell transcriptome states are both inherited, and dynamically reshaped following constrained rules encoded within the cell lineage in basal growth conditions, upon oncogene activation and throughout the process of reversible resistance to therapeutic cues thus adjusting phenotypic output leading to intra-clonal non-genetic diversity.

## Abstract (web summary)

Cellular plasticity is a core biological process; however, observing diversity in non-genetic inheritance and the resulting phenotypic outputs, is challenging. Here the authors develop a non-genetically based tracing technology which can be used to reveal lineage-linked transcriptome plasticity.

## Introduction

Cell plasticity is the fundamental ability of cells to rapidly adapt to environmental cues beyond their genomic makeup^1–4^. This becomes apparent at many levels of biology, ranging from organismal adaptation to unicellular and multicellular organisation^2,5–8^, and is thought to play a key role as an evolutionary avatar across the different kingdoms of life as well as during (patho-)physiological processes in humans^9–12^. However, though a large body of circumstantial evidence supports this view, a formal demonstration suggesting that the plasticity and inheritance of non-genetic molecular features supporting cell adaptation plays a major role in cellular systems remained largely concealed, primarily due to the lack of adequate technologies to query such a phenomenon.

The advent of single-cell technologies^13–24^ has revolutionised the way we understand cellular biology in a fundamental manner, as the intricacies underlying populational behaviour can now be traced to individual cells and, hence, ascribe phenotypes to given molecular identifiers and effectors. Importantly, in recent years, a handful of time-resolved molecular tracing technologies have emerged and have revolutionised the study of fundamental aspects of early development and cancer progression by integrating molecular tracing of individual components such as DNA, RNA and/or proteins thus giving a novel spin to single-cell multimodal analysis^25^. However, the tracing capability of most of the lineage tracing technologies reported to date relies on barcodes encoded in reporter genes which are sequentially integrated into multiple genomic loci, either using lentiviral approaches^26,27^, transposon technologies^28–31^ or by the activity of CRISPR-nucleases^29–35^. Therefore, multiple and divergent genome modifications take place in each individual cell within a population prior to their tracing thus affecting the isogeneity of the system and, as a consequence, potentially altering the non-genetic landscape, including its plasticity and inheritance.

Herein we describe a cell lineage tracing method that relies on a battery of episome-encoded unique molecular identifiers which brings the possibility to build directional lineage trees and study the dynamic inheritance and molecular plasticity of non-genetic traits between cells with comparable unaltered genomes. Our method can be coupled to state-of-the-art single-cell RNA sequencing (scRNA-Seq), enabling the study of transcriptome inheritance and plasticity in basal conditions as well as in response to biological cues. Supported by our approach we show that transcriptome states are readily inherited upon cell division/s but also rewire to generate progeny displaying different transcriptome profiles, highlighting their plasticity to sustain population heterogeneity under stable growing conditions. Notably, we observe that the diversity of transcriptome profiles arising for any given cell is not random but is determined and restricted by their lineage. Overall, we provide evidence supporting the notion that transcriptome states dynamically interconvert and that non-genetic diversity plays a fundamental role in shaping phenotypic output even in somatic, determined, fully differentiated cellular systems.

## Results

### Development of Barcode Decay Lineage Tracing (BdLT-Seq)

**Fig. 1:**
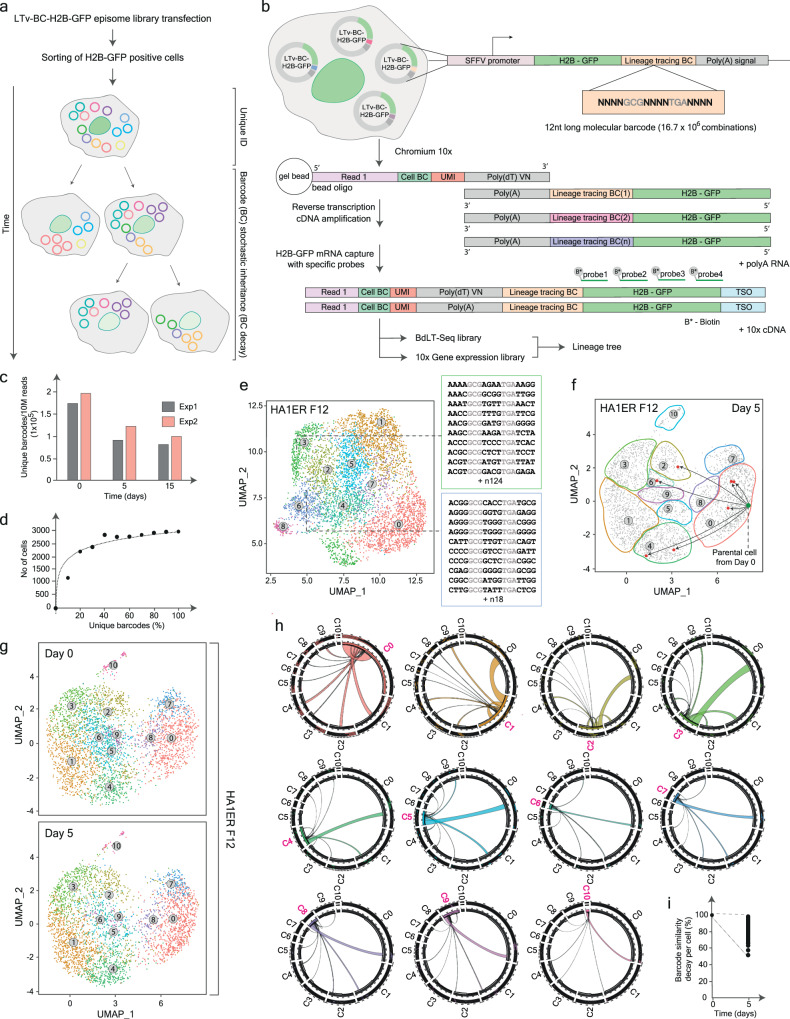
Development of Barcode decay Lineage Tracing (BdLT-Seq) to explore transcriptome plasticity. **a** Scheme displaying BdLT-Seq conceptual framework that can be applied to any given cell system susceptible to transfection with the episomal library (LTv-BC-H2B-GFP lineage tracing episome library) and hence expresses the reporter gene H2B-GFP that is used as a marker to enrich in episome containing cells using fluorescence-activated cell sorting (FACS). Cell ID extraction and lineage tracing deconvolution is depicted as a function of time. **b** Scheme representing the BdLT-Seq vector and the processing of H2B-GFP barcoded 3′UTR within the 10x v3.1 experimental pipeline. **c** Histogram displays the in-bulk total number of unique barcodes per 10 M sequenced reads and its decay at 0, 5, and 15 days of tracing. Data stands for two independent experiments (Exp1 and Exp2) performed in parallel. **d** Scatter plot representing the total number of cells detected as a function of the number of unique barcodes analysed. At 50% barcodes analysed (136,200 unique barcodes) the number of cell IDs traced becomes negligible. **e** UMAP plot depicting scRNA-Seq data for HA1ER F12 clone exemplifying cell ID and the observed transcriptome states. Two cells are individualised and shown together with a subset of their corresponding BdLT-Seq barcode identifiers which provides unequivocal information about cell identity. **f** UMAP plot of scRNA-Seq data obtained from HA1ER F12 clone representing the lineage relationship for a cell at Day 0 (green dot) and its progeny (red dots) at Day 5 of tracing as determined by BdLT-Seq. Gene expression state boundaries are shown as coloured lattices. All cells analysed in the experiment are depicted in grey. **g** UMAP plots of scRNA-Seq data obtained from HA1ER F12 clone displaying cells detected at Day 0 and Day 5 of tracing. **h** Chord diagrams representing transcriptome state dynamics for cells belonging to a particular state/cluster at the beginning of tracing and their divergence after 5 days. Every detected cluster is depicted (C0 to C10) and integrates the collapsed behaviour of all cells that belong to each particular gene expression state. The origin cluster is shown in pink (Day 0) and chords represent the endpoint cluster association (Day 5). **i** Scatter plot depicts barcode similarity decay for all HA1ER cells analysed after 5 days of tracing. Source data are provided as a Source Data file.

**Fig. 2:**
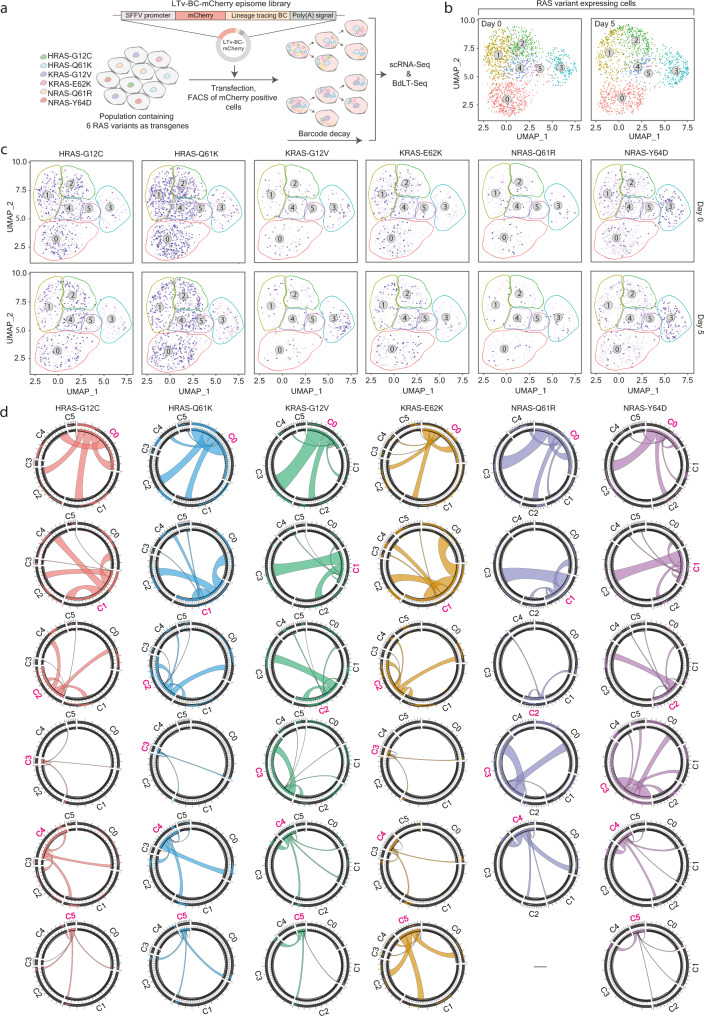
BdLT-Seq reveals RAS variant-specific transcriptome plasticity. **a** Scheme representing the experimental setup for a six-way multiplexed RAS variant (HRAS-G12C, HRAS-Q61K, KRAS-G12V, KRAS-E62K, NRAS-Q61R and NRAS-Y64D) mixed cell population subjected to BdLT-Seq. RAS variants comprised in the cell library are listed. **b** UMAP plots of scRNA-Seq data depicting transcriptome clustering of the RAS variants-mixed population at Day 0 and Day 5 of tracing. **c** UMAP plots of scRNA-Seq data displaying gene expression states identified for each individual RAS variant in the complex mix. Transcriptome states are depicted as coloured lattices and cells belonging to each RAS variant are displayed in blue on Day 0 and Day 5 of tracing. **d** Chord diagrams representing transcriptome state dynamics for the RAS variant multiplexed cell population. All detected clusters are depicted (C0 to C5) and integrate the collapsed behaviour of all cells that belong to a particular gene expression state for each RAS variant. The origin cluster is shown in pink (Day 0) and chords represent the endpoint cluster association (Day 5). Source data are provided as a Source Data file.

**Fig. 3:**
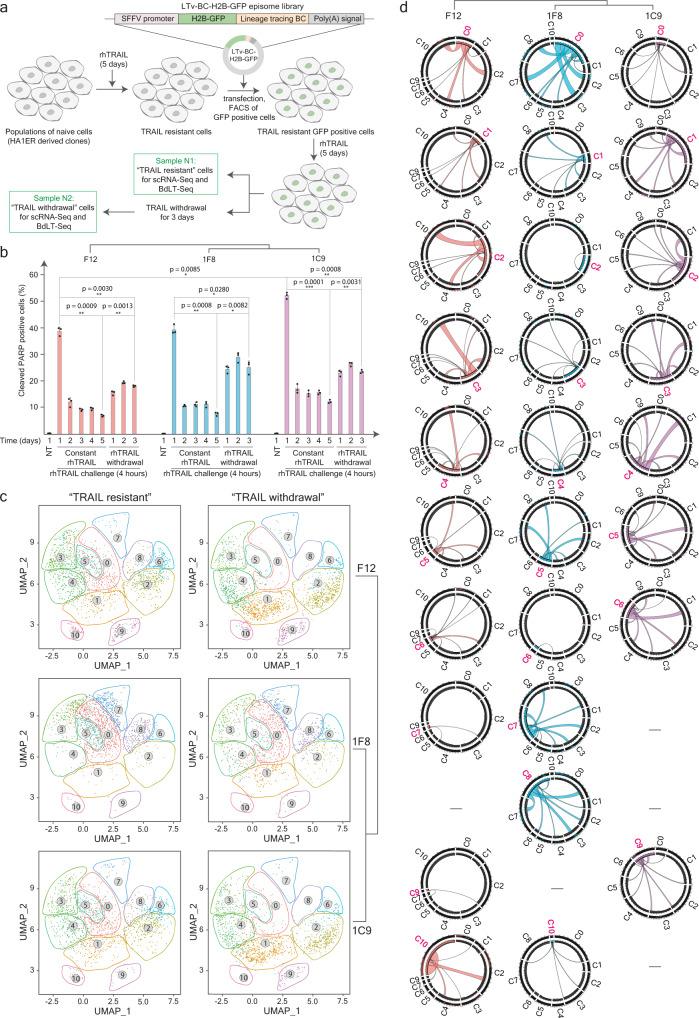
Transcriptome plasticity during transient TRAIL-resistance unveiled by BdLT-Seq. **a** Scheme representing the workflow to generate populations of cells resistant to TRAIL-induced apoptosis (either induced in response to or selected for) and its processing through the BdLT-Seq to explore transcriptome divergence upon TRAIL withdrawal. **b** Histogram depicting TRAIL-induced apoptosis in HA1ER parental clone (F12, pink) and two F12-subclones (1F8 and 1C9, blue and light purple respectively) as determined by cleaved PARP staining analysed by flow cytometry. No TRAIL (NT) and constant rhTRAIL treatment are depicted as controls of acquired TRAIL resistance. Day 1 to Day 3 of rhTRAIL withdrawal is shown to denote escape from TRAIL resistance and reversion to fractional killing induced by TRAIL. Histograms show the mean value ± standard deviation (SD) of three independent biological replicates (~20,000 cells per condition). Statistical significance was assessed by a two-tailed paired Student’s *t*-test. ***P value < 0.0005, **P value < 0.005, *P value < 0.05. The clonal relationship is also depicted. Individual data points are shown. **c** UMAP plots of scRNA-Seq data showing transcriptome states identified for F12, 1F8 and 1C9 cells at Day 0 (TRAIL-resistant, left panel) and Day 3 (TRAIL withdrawal, right panel) of tracing. Gene expression state boundaries are depicted as coloured lattices. The clonal relationship is also depicted. **d** Chord diagrams depicting transcriptome state dynamics for F12, 1F8 and 1C9 cells that belong to a particular state/cluster at Day 0 of tracing (TRAIL-resistant) and their divergence after 3 days (TRAIL withdrawal). All detected clusters are depicted (C0 to C10) and integrate the collapsed behaviour of all cells that belong to each particular gene expression state. The origin cluster is depicted in pink (Day 0) and chords represent endpoint cluster association (Day 3). Source data are provided as a Source Data file.

Our technology is based on the transfection of a library of engineered episomes (LTv-BC-H2B-GFP or LTv-BC-mCherry episome library), each harbouring a unique barcode which is encoded in the 3′ UTR of a reporter gene (either H2B-GFP or mCherry; Figs. 1a, b, 2a, 3a). These high-complexity episomal libraries, that can accommodate up to ~16.7 × 10^6^ unique barcodes, are transfected into cells via standard protocols (Fig. 1a, b and Supplementary Fig. 1a–d). Since almost every episome in our library harbours a unique barcode, the combination of episomes that is introduced into any given cell generates a molecular fingerprint allowing the identification of each cell within a population (Fig. 1a, b, e). The tracing capability of our method relies on two major aspects of episome biology; firstly, EBNA1/OriP based-episomes undergo scheduled replication^36,37^ making these vectors suitable tools for short- and long-term tracing (Fig. 1c and Supplementary Fig. 1e–g). Secondly, though episomal vectors are stably maintained and expressed within transfected cells, they are randomly inherited upon cell division. This barcode partition results in a decay in the number of uniquely barcoded episomes present in cells downstream of any given lineage providing a distinctive barcode-based fingerprint that relates a cell to its cell ancestor, thus allowing to build directional cellular lineage trees (Fig. 1a, c, e, f and Supplementary Fig. 1g). Based on this feature, we termed our technology Barcode decay Lineage Tracing Sequencing (BdLT-Seq).

When transfecting a lineage tracing episomal library (LTv-BC-H2B-GFP episome library) of ~1.2 × 10^6^ unique barcodes in a clonal isogenic cancer cell model (HA1ER cells, F12 clone, Supplementary Fig. 1a, b), we retrieved ~1.7–2 × 10^5^ different barcodes per 10 M reads sequenced at time 0, followed by ~0.9–1.2 × 10^5^ and ~0.8–1 × 10^5^ upon 5 and 15 days of tracing, respectively (Fig. 1c). We confirmed that barcode variability does not impact on episome inheritance as the number of barcodes and their corresponding decay in the overall cell population follows a reproducible pattern, independently of their nature (Fig. 1c). Note that episome replication, as inferred from barcode expression levels, do not overcome episome decay within the experimental timeframes used herein, therefore suggesting that barcode decay can be used to build directional ancestry trees in a robust and reproducible manner (Supplementary Fig. 1e, f, g). Supported by the high reproducibility in barcode decay observed in bulk assays (Fig. 1c), we combined BdLT-Seq with state-of-the-art single-cell technologies (10x Chromium platform) to retrieve the transcriptome of each cell whilst concomitantly assigning branches in their cell-lineage tree (Fig. 1b). This was initially tested in a clonal population of HA1ER cells (F12 clone) which was transfected with a library containing ~1.2 × 10^6^ barcodes, traced for 5 days (Day 0 and Day 5) and subjected to BdLT-Seq-coupled-to-scRNA-Seq analysis. After scRNA-Seq data pre-processing, which includes removal of cells displaying high levels of mitochondrial transcripts, abnormal read counts, cell cycle regression and demultiplexing^38–42^ (Supplementary Fig. 10), we identified up to 10 distinct gene expression clusters and assigned identity to 6012 cells out of the 7032 cells analysed (~85%) across all conditions tested (Fig. 1e). In addition, we verified that neither episome transfection nor reporter expression altered cell cycle progression (Supplementary Fig. 2c, d) or cell clustering based on their transcriptome profiles (Fig. 1g). Furthermore, we evaluated whether cells transfected with BdLT-Seq episomes engage the exogenous DNA recognition/degradation machinery by assessing cGAS/STING pathway activation^43^ (TNF and IL6) (Supplementary Fig. 4) and, though episome degradation or promoter control^44–46^ cannot be ruled out as a mechanism contributing to barcode decay, the reproducibility of our overall results suggest that barcode decay and inheritance is not substantially affected by either of these processes. Importantly, as all timepoints are analysed concomitantly and on a single 10x Chromium run, comparisons between clusters and samples can be done without mathematical integration between datasets. After verifying that the minimal number of barcodes detected in a single run was sufficient and optimal for the experimental conditions tested (Fig. 1d), we built the lineage trees for 191 cells identified at Day 0, for which we traced their progeny to 2362 cells at Day 5 (Fig. 1f–i). Next, we studied the features of each transcriptome state as per that of its ancestor cell by pooling the sequencing data obtained from all cells belonging to a given transcriptome state at Day 0 (cluster C0–C10) and following the lineage of each cell. We observed that though some cells within the progeny display the transcriptome profile of their ancestor cell (i.e., C0-C0), others show divergent transcriptomes which are not random, but restricted to a defined transcriptome repertoire (Fig. 1h and Supplementary Fig. 2e). For example, cells harbouring a transcriptome in C0 generate progeny whose transcriptomes will fall in cluster C0 (potential direct inheritance) but also C1, C2, C3, C4, C5, C7, C8, and C9, but not C6 or C10. On the other hand, cells initially residing in transcriptome state C6 generate progeny whose transcriptomes will diverge to C0, C1, C2, C4, C5, and C9 but not others, at least under the experimental conditions here tested. Furthermore, we verified the potential of BdLT-Seq to retrieve cellular ancestry and its link with transcriptome plasticity upon longer experimental times – other than the ones already tested in HA1ER F12—including timeframes ranging from 0 to 7 days, an intermediate tracing from 7 to 14 days to verify reproducibility, and directly from 0 to 14 days of tracing (Supplementary Figs. 2a, b, 3a, b). As expected, upon lineage reconstruction and transcriptome state annotation, we were able to retrieve 11 transcriptome states displaying transcriptome divergence (Supplementary Figs. 2a, b,  3a, b), supporting our previous observation and validating BdLT-Seq as a tool to explore long-term lineage divergence. In addition, by direct quantification of barcode content by qPCR we determined that barcodes are reliably detected even after a month since the beginning of tracing (Supplementary Fig. 1f, g) suggesting that BdLT-Seq is potentially amenable for experimental settings requiring longer tracing timeframes, which could open the path to in vivo lineage reconstruction. Notably, transcriptome inheritance and restricted transcriptome plasticity within cell lineages were observed not only in the transformed HA1ER F12 clonal cell model but also when studying a clonal population of immortalised cells (HA1E; Supplementary Fig. 5a–d). Together, these results revealed that the transcriptome of an ancestor cell is inherited by its progeny thus perpetuating it in the overall cell population, but it does also diverge into distinct transcriptome states which are somewhat restricted and traceable to a cell lineage, thus fuelling the generation and maintenance of non-genetically heterogeneous cell populations in non-malignant and cancerous settings.

### BdLT-Seq unveils RAS variant-dependent transcriptome plasticity

Multiple lines of evidence suggest that protein members of the RAS family (H-, K- or N-) and its mutant versions facilitate the malignant transformation of cellular systems^47,48^. In that matter, albeit a wealth of information has been gathered for various mutant/oncogenic RAS variants by means of comparing cell systems with divergent genotypes or by addressing the molecular landscape of cells from different tissue of origin^48–50^, a systematic analysis assessing if and how the expression of different RAS oncogenes affects transcriptome plasticity in a variant-dependent manner, a potential key step in cancer onset, has remained unexplored. Thus, we hypothesised that the application of BdLT-Seq coupled to single-cell transcriptome analysis would represent a valuable tool to contribute to fill this void. Therefore, we built a multiplexed cell system in which six mutant RAS variants from the three RAS families (H-, K- and N-) often found in cancer and displaying dissimilar prevalence^51^ (Supplementary Fig. 6a) were individually expressed in an otherwise isogenic clonal population of immortalised HA1E cells (Fig. 2a). Supported by BdLT-Seq-coupled-to-scRNA-Seq, we analysed how transcriptome profiles, heterogeneity, inheritance and plasticity are rewired upon the expression of each of these six RAS variants; concomitantly and in an otherwise genetically comparable cellular population. The analysis of scRNA-Seq data obtained from HA1E cells transduced with the library of six mutant RAS variants retrieved 6 well defined transcriptome clusters within the complex cell mix (Fig. 2b). After cell identification and RAS-variant demultiplexing, we found that, albeit all the six transcriptome clusters were detected in all the 6 RAS mutants studied, an enrichment of individual variants within particular transcriptome states could be readily observed (Fig. 2c). As an example, whilst cells expressing either of the HRAS mutant proteins (HRAS-G12C and HRAS-Q61K) display transcriptomes that fall primarily into C0, C1, C2 and C4, the transcriptome of cells expressing either of the NRAS mutant proteins (NRAS-Q61R and NRAS-Y64D) fall primarily within C3 (Fig. 2c). Interestingly, a clear example of transcriptome divergence driven by the same RAS isoform displaying a different mutation was observed in cells expressing KRAS-G12V which was enriched in C3, whereas cells expressing KRAS-E62K were indeed enriched in C0, C1, C2 and C4. BdLT-Seq-supported cell lineage reconstruction showed that both shared and variant-specific patterns of transcriptome inheritance and plasticity are followed between cells expressing different mutant RAS proteins. For instance, C1 transcriptome inheritance patterns for HRAS-G12C, HRAS-Q61K and KRAS-E62K follow very similar inheritance and plasticity paths (C1, Fig. 2d and Supplementary Fig. 6b), whilst a similar behaviour in transcriptome inheritance and plasticity was also observed between KRAS-G12V, NRAS-Q61R and NRAS-Y64D in C0 (C0, Fig. 2d) and between HRAS-G12C and HRAS-Q61R (C0, Fig. 2d). In contrast, the observed C3 transcriptome plasticity diverges between all the 6 RAS oncogenic mutants (C3, Fig. 2d and Supplementary Fig. 6b). These results support the implementation of our technology in dynamic experimental settings and revealed that the expression of different RAS variants has a distinct impact on lineage-linked transcriptome plasticity; the latter suggesting that the nature and ultimate degree of transcriptome heterogeneity observed in the resulting cell population is, to an extent, dictated by the mutant RAS variant being expressed.

### BdLT-Seq unravels lineage-linked transcriptome dynamics during transient resistance to TRAIL-induced apoptosis

The malignant transformation of cells via the activation of RAS oncogenes has been linked to the acquisition of sensitivity to apoptosis induced by TRAIL (tumour-necrosis factor-related apoptosis-inducing ligand)^52^, a cytokine involved in cancer immunosurveillance and whose therapeutic potential has been evaluated in clinical trials^53^. Importantly, it has been reported that even in clonal populations of cancer cells, only a fraction of the cell population dies upon TRAIL challenge, whereas some cells survive and generate resistant populations while maintained under sustained treatment^54,55^. Interestingly, this resistant population regains sensitivity to TRAIL-induced apoptosis following treatment withdrawal, highlighting the non-genetic basis of such a phenomenon (fractional killing)^54^. Notably, the nature and heterogeneity of transcriptome states supporting the maintenance of non-genetically encoded TRAIL resistance under sustained challenge, as well as the dynamics of transcriptome inheritance and plasticity accompanying the re-acquisition of sensitivity to TRAIL-induced apoptosis have not been elucidated. Thus, we exploited BdLT-Seq-coupled-to-scRNA-Seq to study lineage-linked transcriptome inheritance and plasticity in non-genetically based reversible fractional killing induced by TRAIL in the HRAS-G12V driven HA1ER cell model, in which this phenomenon has been well characterised^52,56^. In line with previous reports, we observed that HA1ER F12 clonal populations establish a resistance to TRAIL-induced cell death following 5 days of treatment and that the re-acquisition of sensitivity to apoptosis is gradually restored upon TRAIL withdrawal, until full reversion is established (Supplementary Figs. 7, 8). To assess whether there is a functional link between the observed transcriptome states present in TRAIL-naive cell populations on the initial sensitivity and transcriptome profiles attained by TRAIL-resistant cells, we subcloned HA1ER F12 parental cells, and selected two subclones (1F8 and 1C9) displaying divergent transcriptome states in basal growing conditions (Supplementary Fig. 9a), but which did not show significant differences in the overall cell morphology, cell cycle progression or proliferation rate when compared to the parental clone F12 (Supplementary Fig. 9b–d). Next, we challenged these three populations with recombinant human TRAIL (rhTRAIL) and, despite displaying rather divergent transcriptome states under basal conditions, all resulted in populations of resistant cells (either induced in response to or selected for) after 5 days of sustained treatment (Fig. 3a, b and Supplementary Fig. 7d). In this context, to explore transcriptome plasticity during TRAIL withdrawal and to capture the complexity of lineage-linked paths determined by founder transcriptome states, resistant populations from all three clones (F12, 1C9 and 1F8) were transfected with the LTv-BC-H2B-GFP episome library whilst maintaining constant TRAIL treatment. Importantly, to maximise our chances to capture divergent transcriptome states between the clones analysed, we postulated that an intermediate time frame between full TRAIL resistance and full reversion would be the most adequate. Therefore, we collected samples at full TRAIL resistance and after 3 days of TRAIL withdrawal and performed BdLT-Seq-coupled-to-scRNA-Seq for all the clones analysed (Fig. 3a, b). Interestingly, as observed in basal growing conditions (Supplementary Fig. 9a), the repertoire of transcriptome states observed in resistant populations from the parental clone F12 and subclone 1C9 display a higher degree of overall similarity when compared to the subclone 1F8, demonstrating a link between the initial transcriptome repertoire and that attained following TRAIL treatment (Fig. 3c). Moreover, BdLT-Seq data robustly retrieved cell lineage trees linked to the transcriptome transitions taking place during TRAIL withdrawal (Fig. 3c, d and Supplementary Fig. 9e) and showed that certain transcriptome states (e.g. C1) display highly similar inheritance paths and plasticity in all three clones, generating progeny that overall populate a similar repertoire of transcriptomes states (i.e. C1 to C2, C3, C4 and C6 in F12, 1F8 and 1C9), whilst also showing some clone-specific diverging patterns (i.e. C1 to C10 only in clone F12; C1 to C8 only in subclone 1F8 and C1 to C0 and C9 only in subclone 1C9) (Fig. 3d). In line with our initial observations (Fig. 1g, h and Supplementary Figs. 2a, b, 3, 5a, b), the differences in plasticity between the transcriptome states can be readily observed within each clone. For example, we found that though C2, C3 and C6 display highly similar plasticity patterns (C2, C3 and C6 populate C1, C2, C3, C4, C6, C9 and C10) within the F12 clone, their potential to generate progeny to C0 and C7 is rather divergent, at least under this experimental setting. Specifically, C3 bears a capacity to populate C0 and C7, whilst C2 does not give rise to cells in those clusters. In contrast, C6 does not generate progeny to C7 while giving rise to cells in C0 upon TRAIL withdrawal (Fig. 3d).

Together, this data supports the implementation of our methodology for studying how non-genetic cell ancestry dictates the inheritance and (degree of) plasticity of individual transcriptomes during highly dynamic processes, revealing its impact on the populational outcome in response to biological and/or therapeutic cues.

## Discussion

Cell plasticity has intrigued researchers for decades as it is prevalent in fundamental processes across the whole of biology^9,10^. Although long acknowledged, only fragmentary circumstantial evidence supports the concept that cell plasticity is a key player in cellular homoeostasis and organismal development and, whilst considered a key component in biological networks, its role as an evolutionary avatar is still debated^7,12,14,57–61^. From the experimental perspective, the observation of metastable states embedded within cell populations at different levels (gene expression, protein levels, among others) has been suggested for many branches of biology ranging from bacteria to multicellular organisms, thus suggesting that indeed phenotypic plasticity could be dynamically linked to its existence^1–4,6,54,62–66^. Notably, plenty of examples support the notion that cell plasticity could underlie phenotypic heterogeneity and, in turn, this could grant cellular systems with a subset of competencies to withstand an ever-changing environment even during the most adverse of conditions^2,3,67–71^. Lately, and with the advent of single-cell genomics (DNA and RNA), a more in-depth analysis has begun to emerge in which metastable states can be readily observed, annotated, and queried. However, primarily due to the genome-altering nature of lineage tracing technologies available^26–35^, little has been done to explore cell plasticity in clonal cell populations where the genetic component remains invariable and even less during non-genetically driven clonal divergence. In that regard, the development of BdLT-Seq enables to fulfil this vacuum in knowledge, as data generated by its means can be used to build a directional lineage tree linking its individual branches to metastable transcriptome states and, therefore, trace the inheritance and reshaping of individual transcriptome states. Importantly, we observed that for a diverse set of conditions (cell types, oncogene activation, drug treatment, etc.), lineage commitment correlates with the initial states observed at a given time and its evolution is directly linked and constrained by it, suggesting that only a limited number of state transitions can be attained for a given condition. This observation suggests the existence of a multiparametric level of organisation somehow encoded in non-genetic networks, and probably limited by the thermodynamic properties of each individual system. It follows that the molecular plasticity underlying phenotypic divergence can be allocated to individual cell states and, as our data suggests, is finite. Bearing this concept in mind, the fact that the expression of a set of oncogenic RAS variants in a given isogenic background promotes transcriptome remodelling following alternative paths suggests that transcriptome plasticity can indeed influence phenotypic output, thus promoting molecular and cellular heterogeneity within the population. Indeed, our data strongly suggest that the genetic component interacts with and/or rewires non-genetic networks to determine phenotypic response to biological cues. However, the non-genetic component seems to determine a restricted set of phenotypic outputs that, as stated above, is lineage-linked and finite.

Interestingly, our data also suggests that barcode decay can be paralleled to a non-genetic clock whose behaviour could be experimentally and mathematically inferred from episome replication and decay rates; information that can then be used to estimate cell replication timings for a given cellular system. Perhaps, future iterations of BdLT-Seq including these parameters may enable to address whether state transitions occur throughout the duration of a cell cycle or if cell division is required for state transitions, and thereby plastic adaptation, to take place. If so, the application of decay-rate calibrated BdLT-Seq would solve a need that theoreticians have long identified^72,73^ and could propel our understanding of cell plasticity transitions on a wider scale with mathematical modelling at its very core.

Finally, it is worth noting that from the biomedical point of view, our observations may be consequential as - as it has been postulated by many - anti-cancer therapy failure could be explained by the activity of non-genetic components somehow altering phenotypic output^4,66,67,74–77^. Given our observations in rather simple biological scenarios, we prompt the idea that transcriptome plasticity and divergence are indeed major players in phenotypic output variation and population dynamics in response to environmental cues in isogenic model systems and thus, we predict to be also applicable to highly complex scenarios such as tumour evolution. It follows that genetic makeup, when used as a biomarker of response/relapse for a given (patho-)physiological condition, unravels only a part of the puzzle and is not informative enough given the plasticity of the non-genetic component and its key role in determining phenotypic output.

## Methods

### Cell lines

Immortalised HA1E (hTERT and SV40ER) and tumourigenic HA1ER cells (hTERT, SV40ER and HRAS-G12V) from stepwise tumourigenesis models generated from normal human embryonic kidney cells were obtained from Dr. Hahn (Broad Institute of MIT and Harvard, Cambridge, USA). Cells were cultured in DMEM (1 g/L glucose) (Gibco, cat. #10567014) supplemented with 10% heat-inactivated foetal calf serum (FCS, Gibco) and 100 μg/ml hygromycin (hTERT selection, Thermo Fisher Scientific, cat. #10687010), 400 μg/ml G418 (SV40ER selection, Thermo Fisher Scientific, cat. #10131035) and 0.5 μg/ml puromycin (HRAS-G12V selection, Thermo Fisher Scientific, cat. #A1113803). Single cells were isolated by Flow cytometry Activated Cell Sorting (FACS, BD FACSAria III; BD Biosciences) and expanded in standard culture conditions to establish clonal populations. LentiX 293 T were obtained from Prof. Lacaud (Cancer Research UK Manchester Institute, Manchester, UK) and cultured in DMEM (1 g/L glucose) (Gibco, cat. #10567014) supplemented with 10% heat-inactivated FCS (Gibco). All cell lines used in this study were plated at a confluency of 1 × 10^6^ cells/6010 mm^2^ surface with 10 ml fresh medium, passed every 48 h and kept in culture for a maximum of 15 passages. All cell lines were grown at 37 °C with 5% carbon dioxide (CO_2_) saturation. Mycoplasma contamination was analysed by reverse transcription followed by quantitative Polymerase Chain Reaction (RT-qPCR) (VenorGeM qEP, cat. #11-9100). The identity of all cell lines used in this study was confirmed using Verogen MainstAY Kit for STR profiling (Qiagen, cat. #V16000142).

### Lineage tracing episome libraries (LTv-BC-H2B-GFP and LTv-BC-mCherry)

GBX – an episomal (EBNA1/OriP) vector was a gift from Linzhao Cheng (Addgene, cat. #64123)^78^ and engineered to build a low-copy propagating vector in bacterial hosts and with the potential to express either the H2B-GFP fusion protein or mCherry in mammalian systems. Briefly, pRB322 (Promega, cat. #D1511) was used to amplify the origin of replication (*bom* and *rop* region) by PCR which was shuttled into the GBX vector to replace the high-copy number origin of replication already present. In parallel, gBlock DNA fragments were used to amplify either the H2B-GFP or mCherry sequence by PCR, then cloned into pGEM-T-easy (Promega, cat. #A1360), and finally shuttled into the previously generated low-copy GBX vector (LTv-H2B-GFP and LTv-mCherry vectors). The sequences of the above-mentioned constructs were verified by Sanger sequencing before proceeding to the next step. Next, the library of lineage tracing plasmids was generated by introducing a molecular barcode into the 3′UTR of the H2B-GFP or mCherry. Briefly, the 3′UTRs of the LTv-H2B-GFP and LTv-mCherry vectors were PCR amplified using a primer that contains a 12 nucleotide long random sequence split into three segments by intervening nucleotides (NNNNGCGNNNNTGANNNN, barcodes, Supplementary Data 1). The amplicons were cloned into the LTv-H2B-GFP or LTv-mCherry vectors using HindIII and XbaI restriction sites (LTv-BC-H2B-GFP and LTv-BC-mCherry vectors). The high-complexity library was propagated using an electrocompetent MegaX DH10B T1 bacteria strain (Invitrogen, cat. #C640003). The final LTv-BC-H2B-GFP lineage tracing episome (8847 bp) library contained ~1.2 × 10^6^ unique barcodes. The final LTv-BC-mCherry lineage tracing episome (8462 bp) library contained ~0.5 × 10^6^ unique barcodes.

### Transfection with the LTv-BC-H2B-GFP and LTv-BC-mCherry episome libraries

Transfection of HA1E and HA1ER cells with the LTv-BC-H2B-GFP and LTv-BC-mCherry lineage tracing episome libraries was performed following standard direct transfection protocol using Lipofectamine LTX reagent (Invitrogen, cat. #153385000). The expression of H2B-GFP and mCherry was monitored by fluorescent microscopy (Thermo Fisher Scientific, cat. #AMEX1000) and flow cytometry (BD LSRFortessa X-20 Cell Analyzer, BD Biosciences) 24- or 48-h post-transfection depending on the experimental setup. Flow cytometry data were analysed using FlowJo software (BD, version 10.8.0).

### In-bulk analysis of barcode decay

HA1ER cells were transfected with the LTv-BC-H2B-GFP episome libraries as described in the section 'Transfection with the LTv-BC-H2B-GFP and LTv-BC-mCherry episome libraries'. Forty-eight hours post-transfection GFP-positive cells were sorted using BD FACSAria III Cell Sorter (BD Biosciences). Cells were expanded in standard culture conditions for 10 days and then split into two samples: sample N1 (Day 0) was subjected to the total RNA extraction. The remaining cells were expanded in standard culture conditions for 5 extra days and then split into two samples: sample N2 (Day 5) was subjected to the total RNA extraction and the remaining cells were expanded in standard culture conditions for 10 extra days followed by total RNA extraction—sample N3 (Day 15). Total RNA was isolated using TRIzol reagent (Invitrogen, cat. #15596-026) following the manufacturer’s instructions. Reverse transcription was performed using QuantiTect Reverse Transcription Kit (Qiagen, cat. #205311) following the manufacturer’s instructions. Sequencing libraries were generated by amplifying the 3′UTR of H2B-GFP using Expand High-Fidelity Polymerase System (Roche, cat. #14676700) and primers that specifically recognise the H2B-GFP sequence surrounding the barcodes, generating standard Illumina paired-end constructs containing P5 and P7. Final sequencing library quality was assessed by Fragment Analyser 5200 (Agilent, cat. #M5310AA). Libraries were sequenced on a NovaSeq 6000 version 1.5 (Illumina, cat. #20013850).

### BdLT-Seq

HA1ER or HA1E cells were transfected (1.2 million cells) with the LTv-BC-H2B-GFP or LTv-BC-mCherry episome libraries as described in section 'Transfection with the LTv-BC-H2B-GFP and LTv-BC-mCherry episome libraries' with an efficiency ranging from 25–35%. Forty-eight hours post-transfection GFP or mCherry positive cells were sorted (50,000 cells) using BD FACSAria III Cell Sorter (BD Biosciences) or BD InFlux Cell Sorter (BD Biosciences) aiming for >94% purity after sorting (Supplementary Fig. 1a–d). Cells were expanded in standard culture conditions for 5 days—Phase I—a barcode decay phase in which most of the episomes are lost upon cell division (Supplementary Fig. 1e–g). The second barcode decay phase—Phase II—begins ~5–7 days after transfection where the episomes seem to stabilise however still display a characteristic episome loss (Supplementary Fig. 1e–g). All BdLT-Seq experiments displayed herein had been performed during Phase II. For that, cells were trypsinised (Invitrogen, cat. #25300104), collected by centrifugation at 230 × *g*, 20 °C, and plated at 2000 to 3000 cells per well (0.32 cm^2^) according to experimental conditions. Five to 6 days later (total number of cells ~80,000) cells were split into two samples: sample N1 (Day 0) was stored in liquid nitrogen (90% FCS, 10% DMSO) and sample N2 (Day 5 or Day 7) was expanded in standard culture conditions for 5 or 7 (depending on the experiment) extra days. Five/seven days later cells were split into two samples: sample N2 (Day 5/Day 7) was stored in liquid nitrogen (90% FCS, 10% DMSO) and sample N3 (Day 14) was expanded in standard culture conditions for 7 extra days and then stored in liquid nitrogen (90% FCS, 10% DMSO). The day before single-cell RNA sequencing the cells were returned to standard culture conditions for 24 h. The following day, samples were processed for scRNA-Seq (10x Genomics Chromium, Single-Cell RNA-Seq System, version 3.1, cat. #PN-1000269) coupled to lineage tracing (BdLT-Seq). Briefly, cells were collected by trypsinisation, washed in 5% FCS PBS, resuspended in 5% FCS PBS, and incubated for 10 min, on ice Hashtag antibodies coupled to a specific oligonucleotide (HTO) (BioLegend, cat. #A0251, A0252, A0253, A0254, A0255, A0256, A0257, A0258, A0259, A0260, clone LNH-94 2M2) were used to specifically label individual samples for multiplexing purposes^38^. For that, cells were incubated with the HTO (10 μg/ml) at a ratio of 0.5 μg per 50,000 cells for 30 min, on ice. Cells were then washed three times using 0.04% BSA PBS and counted using a Neubauer counting chamber (Marienfeld, cat. #0640030). Samples were then combined according to the experimental setting and 20,000 cells (for the expected outcome of 10,000 cells) were loaded onto the 10x Chromium platform (10x Genomics, cat. #1000204). Samples were processed following standard 10x Chromium protocol (10x Genomics, version 3.1, cat. #PN-1000269) up until the cDNA amplification reaction. cDNA amplification reaction was supplemented with the HTO additive primer (Supplementary Data 1) and followed by a cDNA clean-up separating the HTO fraction from the cell cDNA using standard methodologies (Beckman Coulter, AMPure XP, cat. #A63880). All size-selected fractions (cell cDNA and HTO cDNA) were assessed by Fragment Analyser 5200 (Agilent, cat. #M5310AA). Next, HTO-depleted cDNA fraction (cell cDNA) was used to capture H2B-GFP or mCherry cDNA. For that, 5 μl of Dynabeads MyOne Silane beads (Invitrogen, cat. #37002D) were washed twice in 500 μl RLT lysis buffer (Qiagen, cat. #79216), resuspended in 140 μl RLT buffer (3.5x volume of initial sample) and added to 40 μl of the HTO-depleted cDNA fraction. Then 180 μl of absolute ethanol was added to the reaction (4.5x volume of the initial sample), mixed well and incubated for 15 min at room temperature in a ThermoMixer C (300 rpm, Eppendorf, cat. #EP5382000015). The supernatant was discarded, and the beads were washed three times in 80% ethanol, dried for 1–2 min at room temperature, and eluted in 5 μl nuclease-free water (10–15 min at room temperature, ThermoMixer C at 300 rpm). To capture H2B-GFP or mCherry cDNA, a set of four sequence-specific biotinylated probes for either H2B-GFP or mCherry (Supplementary Data 1) was used. The probes were hybridised to the corresponding cDNA using xGen Hybridisation and Wash Kit (IDT, cat. #1080577) following the manufacturer’s instructions. Briefly, 8.5 μl of Gen 2x Hybridisation buffer was combined with 2.7 μl of Gen Hybridisation enhancer, 5 μl cDNA fraction and 2 μl of the probes (4 pmol, pool of four probes), mixed, incubated for 10 min at room temperature, followed by incubation in a thermocycler: 30 s at 95 °C and 16 h at 65 °C (lid temperature 105 °C). Following hybridisation, the H2B-GFP or mCherry cDNA fractions were pulled down using streptavidin beads. Twenty microliters of streptavidin beads were washed twice using 100 μl xGen 1x Bead wash buffer and then resuspended in 17 μl bead resuspension buffer (8.5 μl xGen 2x Hybridisation buffer, 2.7 μl Gen Hybridisation buffer enhancer, 5.8 μl nuclease-free water). Seventeen microliters of beads from the previous step were added to the hybridisation reaction and incubated in a thermocycler at 65 °C for 45 min (lid temperature 70 °C) mixing the reaction every 10 min. After incubation, samples were washed using 100 μl of 65 °C wash buffer 1. The supernatant was transferred into a fresh tube and kept on ice (cDNA for 10x gene expression library—GEx fraction). Beads were then washed by adding 150 μl of 65 °C stringent buffer, mixed well and incubated in a thermocycler for 5 min at 56 °C (lid temperature 70 °C). Next, the supernatant was discarded, and the previous step was repeated twice. Then, 150 μl of wash buffer 1 was added to the beads, incubated for 2 min at room temperature and the supernatant was discarded. Following 150 μl of wash buffer 2 was added to the beads and incubated for 2 min at room temperature. After discarding the supernatant, 150 μl of wash buffer 3 was added to the beads and incubated for 2 min at room temperature. To elute H2B-GFP or mCherry cDNA from the beads, 100 μl of denaturation buffer (100 mM NaOH, 0.1 mM EDTA) was added to the complex beads/cDNA and incubated for 15 min at room temperature in a ThermoMixer C (300 rpm). The supernatant was transferred into a fresh tube and 10 μl of 1 M Tris buffer (pH 7.5) supplemented with 10 μl of 1 M HCl was added. Both H2B-GFP and mCherry cDNA fractions (lineage tracing fraction) as well as the fraction that contained the rest of the cDNA for the 10x gene expression library (GEx fraction) were purified using Dynabeads MyOne Silane beads (Invitrogen, cat. #37002D) following the protocol described above. 10x GEx libraries were generated using 120 ng of the cDNA fraction following 10x Chromium protocol (Fragmentation, End repair and Library construction steps). H2B-GFP, mCherry and HTO libraries were generated by amplifying either H2B-GFP, mCherry or HTO cDNA using primers that specifically recognise H2B-GFP, mCherry or HTO on the 5′-end and Read 1 on the 3′-end, generating standard Illumina paired-end sequencing constructs containing P5 and P7 (Supplementary Data 1). Final sequencing library quality was assessed by Fragment Analyser 5200 (Agilent, cat. #M5310AA). Libraries were sequenced on a NovaSeq 6000 version 1.5 (Illumina, cat. #20013850). Sequencing depth: GEx library—35,000−50,000 read pairs per cell, HTO library—2000−3000 read pairs per cell, GFP and mCherry library—10,000−12,000 read pairs per cell. Sequencing type: paired-end, dual indexing. Sequencing reads: Read 1–28 cycles, i7 Index—10 cycles, i5 Index—10 cycles, Read 2—90 cycles.

### Reverse transcription followed by qPCR

HA1ER cells were transfected with the LTv-BC-H2B-GFP episome libraries as described in the section 'Transfection with the LTv-BC-H2B-GFP and LTv-BC-mCherry episome libraries'. Forty-eight hours post-transfection H2B-GFP-positive cells were sorted using BD FACSAria III Cell Sorter (BD Biosciences, at least 94% purity). For direct measurement of barcode decay, cells were expanded in standard culture conditions for 10 days (corresponds to Day 0 in all BdLT-Seq experiments shown herein), and then either collected for RNA extraction or left in standard culture conditions and collected every week for a period of 5 weeks for RNA extraction (Supplementary Fig. 1g). To evaluate episome stabilisation, sorted cells were expanded in standard culture conditions for a week, and then either collected for RNA extraction (Week 1) or sorted to enrich in H2B-GFP-positive cells and further expanded in standard culture conditions for an additional week. This procedure was repeated for 5–6 weeks (Supplementary Fig. 1e, f).

Total RNA was isolated using TRIzol (Invitrogen, cat. #15596-026) following the manufacturer’s instructions. Reverse transcription was performed using QuantiTect Reverse Transcription Kit (Qiagen, cat. #205311) following the manufacturer’s instructions. qPCR was performed using PowerUp SYBR Green Master Mix (Invitrogen, cat. #A25742) in a QuantStudio3 Real-Time PCR System (Thermo Fisher Scientific, cat. #A28136). Data analysis was conducted using Real-Time qPCR Connect Data Analysis Tool (Thermo Fisher Scientific Connect Platform).

### TRAIL treatment and generation of TRAIL-resistant cells (either induced in response to or selected for)

To generate cell populations resistant to TRAIL-induced apoptosis, HA1ER-derived clone F12 and subclones 1F8 and 1C9 were challenged with recombinant human TRAIL (rhTRAIL, 1 μg/ml, R&D Systems, cat. #375-LT-010) for five consecutive days. Briefly, cells were washed with a pre-warmed medium at 37 °C and challenged with rhTRAIL. Four hours later, cells were either collected, fixed and permeabilised with ice-cold methanol for further downstream analysis (cleaved PARP labelling; see below) or left in culture in the presence of rhTRAIL to generate resistant populations. TRAIL-induced apoptosis was determined daily by assessing the percentage of cleaved PARP-positive cells using flow cytometry (BD LSRFortessa X-20 Cell Analyzer, BD Biosciences). Briefly, ice-cold methanol-fixated cells were washed once with 1x PBS followed by incubation in a blocking solution (0.5% BSA PBS) for 1 h at 20 °C. Immunolabelling of cleaved PARP was performed using fluorescently labelled Cleaved PARP Alexa 647 antibody (Cell Signalling, cat. #6987) (1:200 dilution) for 1 h at 20 °C. Once resistance to TRAIL-induced apoptosis was established in the population, cells were transfected with the lineage tracing episome library (LTv-BC-H2B-GFP) as described in section 'Transfection with the LTv-BC-H2B-GFP and LTv-BC-mCherry episome libraries'. Forty-eight hours post-transfection GFP-positive cells were sorted using BD AriaIII Cell sorter (BD Biosciences) and expanded in standard culture conditions in the presence of rhTRAIL for five more days. On day 5, cells were trypsinised, collected by centrifugation, plated at a confluency of 2000 cells per well (0.32 cm^2^) and grown for 5 extra days with constant rhTRAIL. Following this, cells were split into two samples: sample N1 (TRAIL-resistant) containing TRAIL-resistant cells was collected and stored in liquid nitrogen (90% FCS, 10% DMSO) until further processing; sample N2 (TRAIL withdrawal) was cultured in the absence of rhTRAIL for three days and gave rise to the population of TRAIL withdrawn cells. Forty-eight hours later N1 (TRAIL-resistant) samples were thawed and cultured in standard conditions for 24 h in the presence of rhTRAIL (1 μg/ml). The following day, BdLT-scRNA-Seq was performed as described in section 'BdLT-Seq'. Re-establishment of sensitivity to TRAIL-induced apoptosis during TRAIL withdrawal was monitored daily and assessed as the percentage of cleaved PARP-positive cells using flow cytometry (BD LSRFortessa X-20 Cell Analyzer, BD Biosciences).

### Annexin V labelling

Cells were plated at a confluency of 1 × 10^6^ cells/6010 mm^2^ surface. Twenty-four hours later, HA1ER-derived clone F12 and subclones 1F8 and 1C9 were challenged with recombinant human TRAIL (rhTRAIL, 1 μg/ml, R&D Systems, cat. #375-LT-010) for 4 h upon which cells were collected, washed once with 1x PBS, labelled with Annexin V antibody (Invitrogen, cat. # A35108) following manufacturer’s instructions and analysed by flow cytometry (BD LSRFortessa X-20 Cell Analyzer, BD Biosciences).

### SubG1 analysis

Cells were plated at a confluency of 1 × 10^6^ cells/6010 mm^2^ surface. Twenty-four hours later, HA1ER-derived clone F12 and subclones 1F8 and 1C9 were challenged with recombinant human TRAIL (rhTRAIL, 1 μg/ml, R&D Systems, cat. #375-LT-010) for 16 h upon which cells were collected, fixed and permeabilised with ice-cold methanol. SubG1 fraction was assessed using DAPI DNA counterstain (BioTechne, cat. #5748) and analysed by flow cytometry (BD LSRFortessa X-20 Cell Analyzer, BD Biosciences).

### Cell cycle analysis

Cells were plated at a confluency of 1 × 10^6^ cells/6010 mm^2^ surface. Twenty-four hours later, cells were collected, fixed and permeabilised with ice-cold methanol. Cells were stained using DAPI nuclear counterstain (BioTechne, cat. #5748) and cycle progression was assessed by flow cytometry (BD LSRFortessa X-20 Cell Analyzer, BD Biosciences).

### Confluency analysis

Cells were plated at a confluency of 50,000 cells/960 mm^2^ surface. As soon as cells re-attached, the confluency was assessed using IncuCyte S3 Life Cell Analysis Instrument (Sartorius, cat. #4647) imaging plates every 3 h for 4 consecutive days. Confluency analysis was performed using IncuCyte version 2020 C Rev1 software.

### Bright field imaging

Cells were plated at a confluency of 1 × 10^6^ cells/10 cm Petri dishes (6010 mm^2^ surface). Twenty-four hours later, cells were imaged using an inverted microscope (Thermo Fisher Scientific, cat. #AMEX1000).

### Preparation of chemo-competent bacteria

Liquid culture of the bacteria strain SURE2 (Agilent Technologies, cat. #200152) or One Shot ccdB Survival 2 T1 (Invitrogen, cat. #A10460) was set up from a single colony or from frozen glycerol stock in 20 ml of antibiotic-free Luria-Bertani (LB) medium and incubated at 37 °C in an incubator shaker (Infors, HT Multitron, cat. #2292113-18) at 200 rpm overnight. The next day, 10 ml of the bacteria culture was added to 100 ml LB and incubated at 37 °C in an incubator shaker at 200 rpm until an OD_600_ = 0.48 was reached. The bacterial culture was chilled on ice for 20 min before centrifugation at 4 °C at 1000×*g* for 10 min. The supernatant was discarded, and bacterial pellets were resuspended and pooled in 30 ml ice-cold TFBI (100 mM rubidium chloride, 64.3 mM manganese (II) chloride, 30 mM potassium acetate, 10 mM calcium chloride, 15% glycerol, adjusted to pH 5.8). After incubation on ice for 90 min, bacterial suspension was centrifuged at 4 °C at 900 × *g* for 10 min. The supernatant was discarded, and the bacterial pellet was resuspended in 4 ml ice-cold TFBII (10 mM rubidium chloride, 14 mM MOPS, 75 mM calcium chloride, 15% glycerol, adjusted to pH 7.0). Chemo-competent bacteria were aliquoted and flash-frozen in liquid nitrogen before long-term storage at −80 °C.

### Transformation of chemo-competent bacteria

Frozen chemo-competent bacteria were thawed on ice for ~10 min and either purified plasmid DNA or a ligation reaction product was added to the bacteria and gently mixed. After incubation on ice for 30 min, a heat shock at 42 °C for 45 s was applied. Following incubation on ice for 5 min, 1 ml of pre-warmed SOC medium (Invitrogen, cat. #15544034) was added and cells were allowed to recover at 37 °C in an incubator shaker (Infors, HT Multitron, cat. #2292113-18) at 225 rpm for 1 h. Bacteria were collected by centrifugation at 400–600×*g* and resuspended in 100 μl LB medium. Serial dilutions were plated onto LB agar plates containing 50 μg/ml ampicillin for selection of successfully transformed bacteria and incubated either at 30 °C (One Shot ccdB Survival 2 T1 strain) or 37 °C (SURE 2 strain) overnight.

### Preparation of electrocompetent bacteria

Using a single colony or a frozen glycerol stock, a liquid culture of the bacterial strain One Shot ccdB Survival 2 T1 (Invitrogen, cat. #A10460) or MegaX DH10B T1 (Invitrogen, cat. #C640003) was set up in 100 ml antibiotic-free LB medium and incubated at 37 °C in an incubator shaker (Infors, HT Multitron, cat. #2292113-18) at 200 rpm overnight. The following day, 10 ml of the overnight culture was added to 200 ml pre-warmed LB medium and grown at 37 °C in an incubator shaker (Infors, HT Multitron, cat. #2292113-18) at 200 rpm until an OD_600_ = 0.5 was reached. The bacterial suspension was chilled on ice for 20 min and centrifuged at 4 °C at 2200 × *g* for 15 min. The supernatant was discarded, and the bacterial pellet was resuspended in 200 ml ice-cold 10% glycerol. After centrifugation, the supernatant was discarded, and the bacterial pellet was resuspended in 50 ml ice-cold 10% glycerol. The bacterial suspension was spun-down, the supernatant was discarded, and the pellet was resuspended in 8 ml ice-cold 10% glycerol. After final centrifugation, the bacterial pellet was resuspended in 600 μl ice-cold 10% glycerol and directly used for electroporation or stored at −80 °C.

### Transformation of electrocompetent bacteria

Either purified plasmid DNA, ligation or recombination reactions was added to the electrocompetent bacteria and carefully mixed. Following a 1-min incubation on ice, the suspension was transferred to a pre-chilled 0.1 cm electroporation cuvette (Bio-Rad, cat. #1652089) and electroporated using Ec1 programme on MicroPulser Electroporator (Bio-Rad, cat. #1652100). Following this, 1 ml SOC medium was added to the cuvette and transferred to a microcentrifuge tube. For recovery, bacteria were incubated at 37 °C in an incubator shaker (Infors, HT Multitron, cat. #2292113-18) at 225 rpm for 1 h. Following collection using centrifugation at 400–600 × *g*, bacteria were resuspended in LB medium and serial dilutions were plated onto LB agar plates containing 50 mg/ml ampicillin and were incubated at 30 or 37 °C overnight depending on the bacterial strain used.

### Generation of the lentiviral library of six RAS variants with allocated molecular barcodes in their H2B-GFP reporter

A gBlock DNA fragment encoding the H2B-GFP fusion containing a NdeI site in the 3′UTR and matching restriction sites at the ends for further downstream cloning into pLEX_307 (gift from David Root; Addgene, cat. #41392) (KpnI and SbfI) was amplified by PCR and cloned into the pGEM-T Easy vector system. Following verification of the construct by Sanger sequencing, this H2B-GFP fragment was further subcloned into the pLEX_307 vector via One Shot ccdB Survival 2 T1 bacteria strain (Invitrogen, cat. #A10460). To introduce a molecular barcode into the 3′UTR of the H2B-GFP gene, a gBlock DNA fragment with compatible restriction sites (NdeI and SbfI) was amplified by PCR using a primer with 12 random nucleotide barcodes split into three segments by intervening nucleotides (NNNNGCGNNNNTGANNNN) (Supplementary Data 1), and further cloned into the pGEM-T Easy vector. Pooled plasmid DNA purification was performed (Qiagen Plasmid Midi Kit cat. #12143), and the plasmid mix was digested using NdeI and SbfI for sub-cloning the barcoded fragment into the previously generated pLEX-H2B-GFP plasmid, and in-lab generated electrocompetent One Shot ccdB Survival 2 T1 bacteria were transformed. Approximately 50,000 bacteria colonies were pooled for plasmid DNA purification of the barcoded pLEX-H2B-GFP plasmid mix. RAS variants were obtained from the Target Accelerator Pan-Cancer Mutant Collection (Addgene, cat. #1000000103)^47^. Individual liquid cultures of bacteria expressing pDONR plasmids encoding each of the six RAS variants included in our assay were pooled according to their OD_600_ to achieve a balanced representation, and then pDONR-RAS plasmid mix was purified using Qiagen Plasmid Midi Kit (cat. #12143). Following this, the pDONR-RAS plasmid mix was used to shuttle the oncogene coding sequences into the previously generated pLEX-H2B-GFP plasmid mix by recombination using Gateway LR Clonase II Plus enzyme (Invitrogen, 12538120) following manufacturer’s instructions. A unique molecular barcode was assigned to each RAS variant by Sanger sequencing (Supplementary Data 1) the barcode region in the 3′UTR of H2B-GFP as well as the RAS variant region. Finally, liquid cultures of the bacteria clones containing the six final lentiviral transfer vectors (pLEX-H2B-GFP-BC-RAS: HRAS-Q61K, HRAS-G12C, KRAS-G12V, KRAS-E62K, NRAS-Q61R and NRAS-Y64D) were pooled based on their optical density and plasmid DNA was purified, rendering the transfer vector mix for lentivirus production. For that, transfection of LentiX 293 T cells with RAS transfer vector mix, packaging vector (psPAX2, Addgene, #12260) and envelope vector (pMD2.G, Addgene, #12259) was performed following standard transfection protocols using Lipofectamine LTX reagent (Invitrogen, 153385000).

### Determination of the functional lentiviral titre

Increasing volumes of lentivirus stocks (0–500 μl) were added to 150,000 cells and supplemented with polybrene (Sigma, cat. #H9268-5G) to a final concentration of 2 μg/ml. The percentage of GFP-positive cells was determined 48 h post-transduction by flow cytometry (BD LSRFortessa X-20 Cell Analyzer, BD Biosciences) and FlowJo software (BD, version 10.8.0) was used for data analysis. The functional viral titre, calculated as the number of viral particles (i.e. transducing units (TU)) capable to transduce a particular cell line per volume was calculated using the following formula:

$${{{{{\rm{functional\,titer}}}}}} \, \left[\frac{{{{{{\rm{TU}}}}}}}{{{{{{\rm{\mu }}}}}}{{{{{\rm{l}}}}}}}\right]=\frac{{{{{{\rm{cell}}}}}} \, {{{{{\rm{number}}}}}} \, {{{{{\rm{x}}}}}} \, {{{{{\rm{frequency}}}}}} \, {{{{{\rm{of}}}}}} \, {{{{{\rm{GFP}}}}}}+{{{{{\rm{cells}}}}}} \, [\%]}{{{{{{\rm{volume}}}}}} \, {{{{{\rm{of}}}}}} \, {{{{{\rm{virus}}}}}} \, [{{{{{\rm{\mu }}}}}}{{{{{\rm{l}}}}}}]}$$

### BdLT-Seq in a multiplexed cell population expressing RAS variants

HA1E cells were transduced with a pooled RAS lentiviral library (six RAS variants) at a multiplicity of infection (MOI) of 0.05 (5%) supplemented with polybrene at a final concentration of 2 μg/ml. Forty-eight hours post-transduction, GFP-positive cells were sorted and collected by FACS (BD InFlux Cell Sorter, BD Biosciences) and re-plated in standard growing conditions. After 4 days, cells were transfected with the LTv-BC-mCherry episome library following the protocol described in the section 'Transfection with the LTv-BC-H2B-GFP and LTv-BC-mCherry episome libraries'. The growth medium was replaced the next day and cells were harvested 48 h post-transfection. GFP-mCherry double positive cells were collected by FACS (BD InFlux Cell Sorter), expanded for 5 extra days, and then plated in standard culture conditions at 3000 cells per well (0.32 cm^2^) and expanded following the protocol described in section 'BdLT-Seq' up until GFP and mCherry cDNA capturing step. At this stage, the HTO-depleted cDNA fraction (10 μl) was split into two samples: sample N1 was used for GFP cDNA capturing (RAS-variant identification) and sample N2 was used for mCherry cDNA capturing (lineage tracing).

### Data analysis and pipelines

10x Chromium single-cell data was analysed using Seurat v4.0^39^. HTO-labelled^38^ cells were demultiplexed using the HTODEMUX module of Seurat and further processed for quality control and cell filtering taking into account nFeature_RNA, nCount_RNA and mitochondrial content (Supplementary Fig. 10). Further pre-processing included the determination of the expression of cell cycle-related genes and mathematical regression to remove cell cycle-related bias in cluster processing^41,42^. Similarly, variance in cluster detection due to high levels of mitochondrial transcripts present per cell was also regressed prior to final clustering. HTO and BdLT-Seq data were processed using CITE-seq-count^40^ prior to demultiplexing or barcode counting. BdLT-Seq barcode reads were pre-processed to remove sequencing artefacts, flanking and intervening sequences using cutadapt v3.7^79^. Clean barcodes were then processed using CITE-seq-count to generate lineage tracing files. A custom Node script was used to determine lineage relationships based on a similarity score taking into account the number of similar barcodes between two cells and data obtained by these means was used to build a directional cell lineage tree (Supplementary Code). Lineage visualisation was generated using CIRCOS^80^. All data analysed across the manuscript was pre-processed/processed using R (v4.2.0) scripts. Final image processing and output was obtained using Adobe Illustrator.

### Statistics and reproducibility

All histograms displayed in the manuscript show mean value ± standard deviation (SD) of two/three independent biological replicates. Statistical significance was assessed by a two-tailed paired Student’s *t*-test. ***P value <0.0005, **P value <0.005, *P value <0.05.

As part of scRNA-Seq data pre-processing, cells found to be displaying multiple HTO (hashtag oligo) labelling after demultiplexing were excluded from further analysis. Moreover, to attenuate artefactual clustering, cells displaying high levels of mitochondrial transcripts and abnormal read counts were masked. Further processing included the mathematical regression of the datasets based on cell cycle-related genes expression to remove cell cycle-related bias in cluster mapping. Random sampling was applied to BdLT-Seq data libraries to optimise the number of barcodes needed to build lineage trees (Fig. 1d) and to reduce the computational burden.

All attempts of replication were successful. No statistical method was used to predetermine the sample size. No samples were excluded from the analysis. The experiments were not randomised and the investigators were not blinded during experiments and outcome assessment.

### Reporting summary

Further information on research design is available in the Nature Portfolio Reporting Summary linked to this article.

## Supplementary information

Supplementary Information

Description of Additional Supplementary Files

Supplementary Data 1

Supplementary Code

Reporting Summary

## Data Availability

All data used to generate figures in this study is provided as Source Data. Sequencing data have been deposited in the Gene Expression Omnibus database under the accession code “GSE223496.” Source data are provided with this paper.

## Source data

Source data

## Code availability

All data were analysed using publicly available, benchmarked, and validated pipelines (R v4.2.0, Seurat v4.0, CITE-seq-count and cutadapt v3.7). The data analysis rationale is described in the Methods section and illustrated in Supplementary Fig. 10. A custom Node script used to determine barcode similarity between data points used to reconstruct lineage relationships is provided as Supplementary Code. For any data analysis-related inquiries, please contact MP (Maximiliano.Portal@cruk.manchester.ac.uk).
