# Supplementary material for: BdLT-Seq as a barcode decay-based method to unravel lineage-linked transcriptome plasticity
Source: Nat Commun. 2023 Feb 25;14:1085. doi: 10.1038/s41467-023-36744-1 (PMC9968323; doi:10.1038/s41467-023-36744-1)
Supplement: Supplementary file 1 — Supplementary Information [file 41467_2023_36744_MOESM1_ESM.pdf]

Supplementary Information

BdLT-Seq as a barcode decay-based method to unravel lineage-linked transcriptome plasticity

Yelyzaveta Shlyakhtina[1], Bianca Bloechl[1] and Maximiliano M. Portal[1,*]

[1] Cell Plasticity & Epigenetics Lab, Cancer Research UK – Manchester Institute, The University of Manchester, SK10 4TG, Manchester, United Kingdom

* Corresponding author (email: Maximiliano.Portal@cruk.manchester.ac.uk)

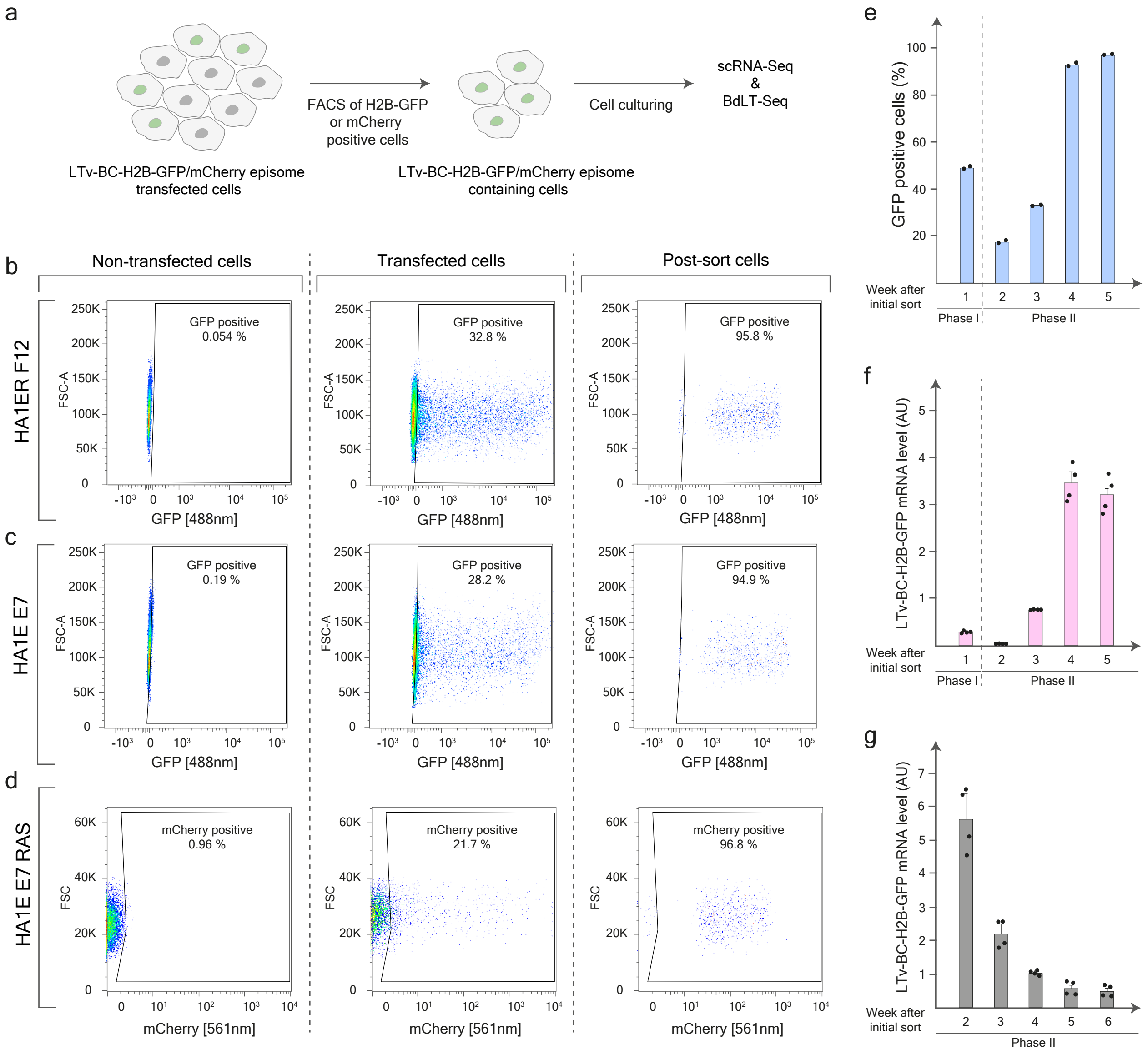

Supplementary Figure 1. Experimental set-up for BdLT-Seq. a- Scheme representing the workflow to generate populations of cells enriched in H2B-GFP or mCherry expressing cells for BdLT-Seq experiments. **b-d-** Scatter plots obtained from flow cytometry assays assessing the percentage of H2B-GFP (b and c) or mCherry (d) positive cells in non-transfected, LTv-BC-H2B-GFP or LTv-BC-mCherry episome libraries transfected populations (LT episome transfected cells) and populations after FACS of H2B-GFP (b and c) or mCherry (d) positive cells in HA1ER (b), HA1E (c) and HA1E-RAS (d) cells. Scatter plots from representative experiments are shown. Percentage of H2B-GFP or mCherry positive cells is indicated. **e-** Histogram depicting the percentage of H2B-GFP positive cells as determined by flow cytometry, beginning one week after and for a total of 5 weeks upon post-transfection sorting. Cells were sorted weekly using FACS to enrich in H2B-GFP positive cells followed by one week in standard culture conditions upon which the percentage of H2B-GFP positive cells was analysed by flow cytometry. Data is represented as the fraction of H2B-GFP positive cells (%). Histograms show mean value of two independent biological replicates (~20,000 cells per condition). Individual data points are depicted. **f-** Reverse transcription followed by qPCR analysis of H2B-GFP mRNA levels in LTv-BC-H2B-GFP episome transfected HA1ER cells one week after FACS sorting of H2B-GFP positive cells for a total of 5 weeks after initial post-transfection normalized to Glyceraldehyde 3-phosphate dehydrogenase (GAPDH) levels. Cells were sorted weekly using FACS to enrich in H2B-GFP positive cells followed by one week in standard culture conditions upon which the percentage of H2B-GFP positive cells was analysed by flow cytometry. Histograms represent the mean value +/- propagated error of two independent biological replicates (two technical replicates each). Individual data points are depicted. **g-** Reverse transcription followed by qPCR analysis of H2B-GFP mRNA levels in LTv-BC-H2B-GFP episome transfected HA1ER cells at 2, 3, 4, 5 and 6 weeks after initial post-transfection sorting normalized to Glyceraldehyde 3-phosphate dehydrogenase (GAPDH) levels. Histograms represent the mean value +/- propagated error of two independent biological replicates (two technical replicates each). Individual data points are depicted. Source data are provided as a Source Data file.

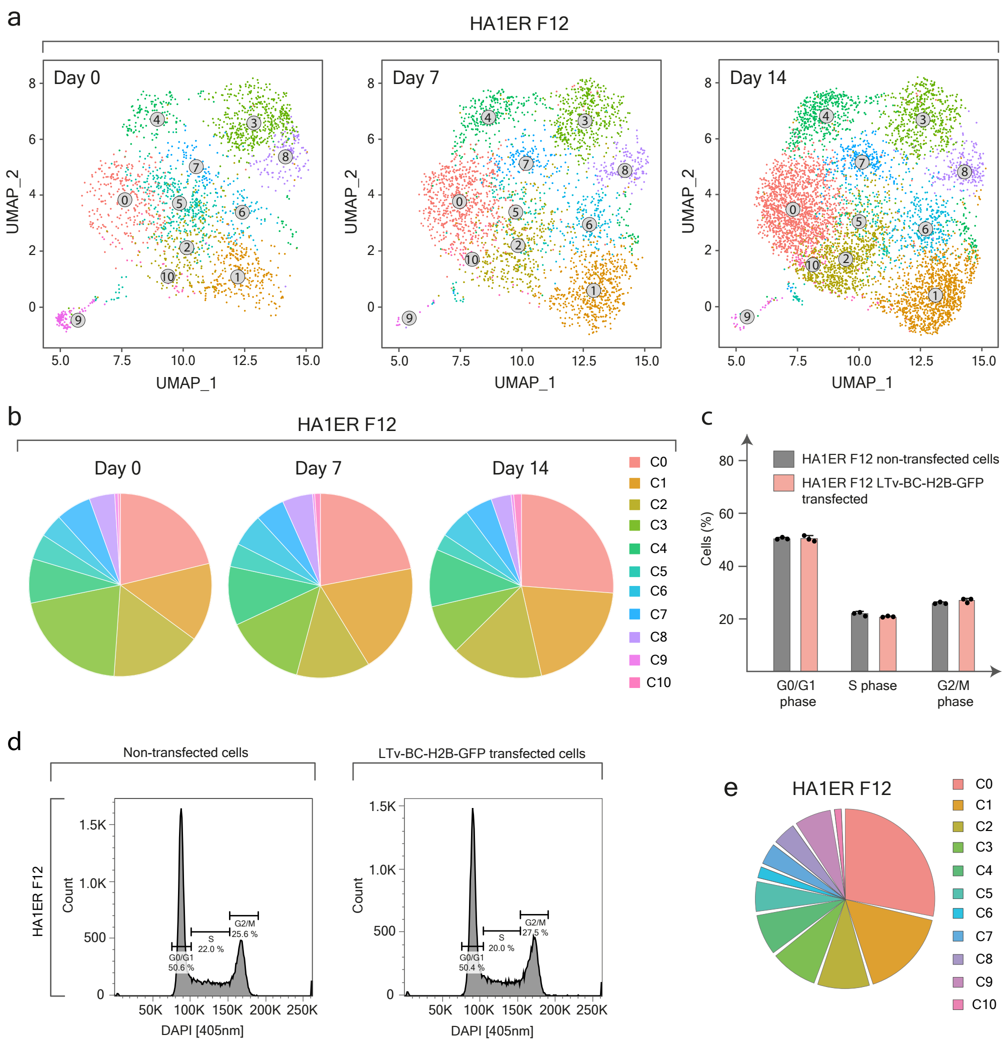

Supplementary Figure 2. scRNA-Seq revealed that clonal population of HA1ER cells display multiple transcriptome states in standard growing conditions. **a-** UMAP plots of single-cell RNA sequencing data depicting gene expression states of HA1ER F12 cells are shown. Samples were obtained and processed at Day 0, Day 7 and Day 14. **b-** Pie charts representing the number of cells contributing to each gene expression state identified. Note that the proportion of cells in predominant clusters remain constant over time. **c-** Histogram showing the representation of non-transfected and LTv-BC-H2B-GFP-transfected HA1ER F12 cells throughout the cell cycle as analysed by DAPI staining using flow cytometry. Histograms show mean value +/- standard deviation (SD) of three independent biological replicates (~30,000 cells per condition). Individual data points are depicted. **d-** Histograms show the representation of cells in each cell cycle phase for HA1ER non-transfected or LTv-BC-H2B-GFP episome transfected cells as analysed by DAPI staining using flow cytometry. Images from one representative experiment are shown. Percentage of cells in different phases of cell cycle is indicated. **e-** Pie charts displaying the transcriptome state distribution of cells traced by BdLT-Seq for HA1ER F12 matching Fig. 1h. Source data are provided as a Source Data file.

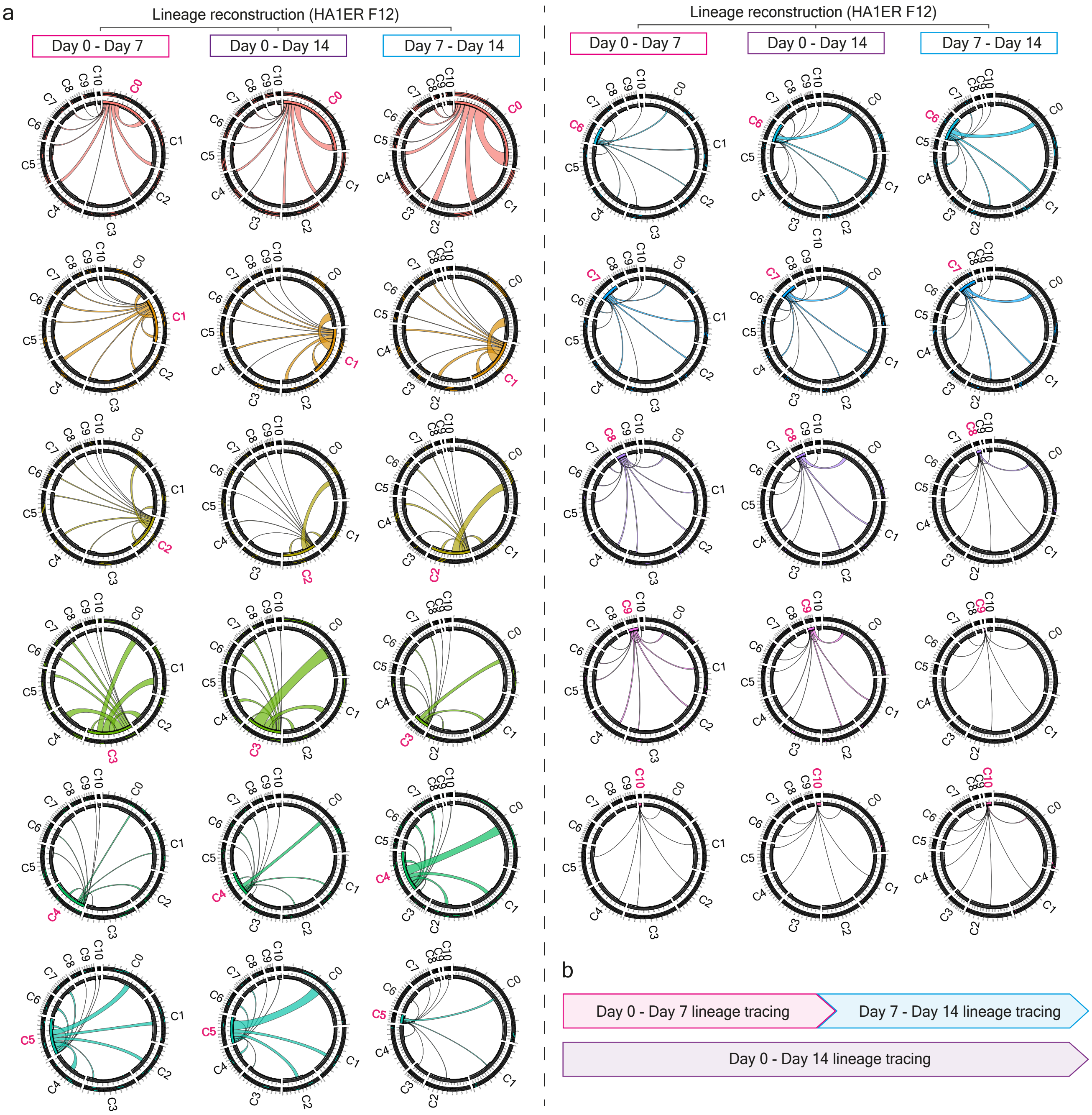

Supplementary Figure 3. BdLT-Seq unravels sequential transcriptome state transitions during long-term tracing experiments. a- Chord diagrams depicting transcriptome state dynamics for HA1ER F12 cells (Supplementary Fig. 2a) that belong to a particular state/cluster at Day 0 and Day 7 of tracing and their divergence after 7 days (Day 7 and Day 14) - Day 0 - Day 7 and Day 7 - Day 14, respectively, or 14 days (Day 14) - Day 0 - Day 14. All detected clusters are depicted (C0 to C10) and integrate the collapsed behaviour of all cells that belong to each particular gene expression state. Origin cluster is depicted in pink (at Day 0 or Day 7) and chords represent end point cluster association (Day 7 or Day 14). **b-** Scheme representing the workflow to perform long-term lineage tracing experiments (0 to 14 days). Source data are provided as a Source Data file.

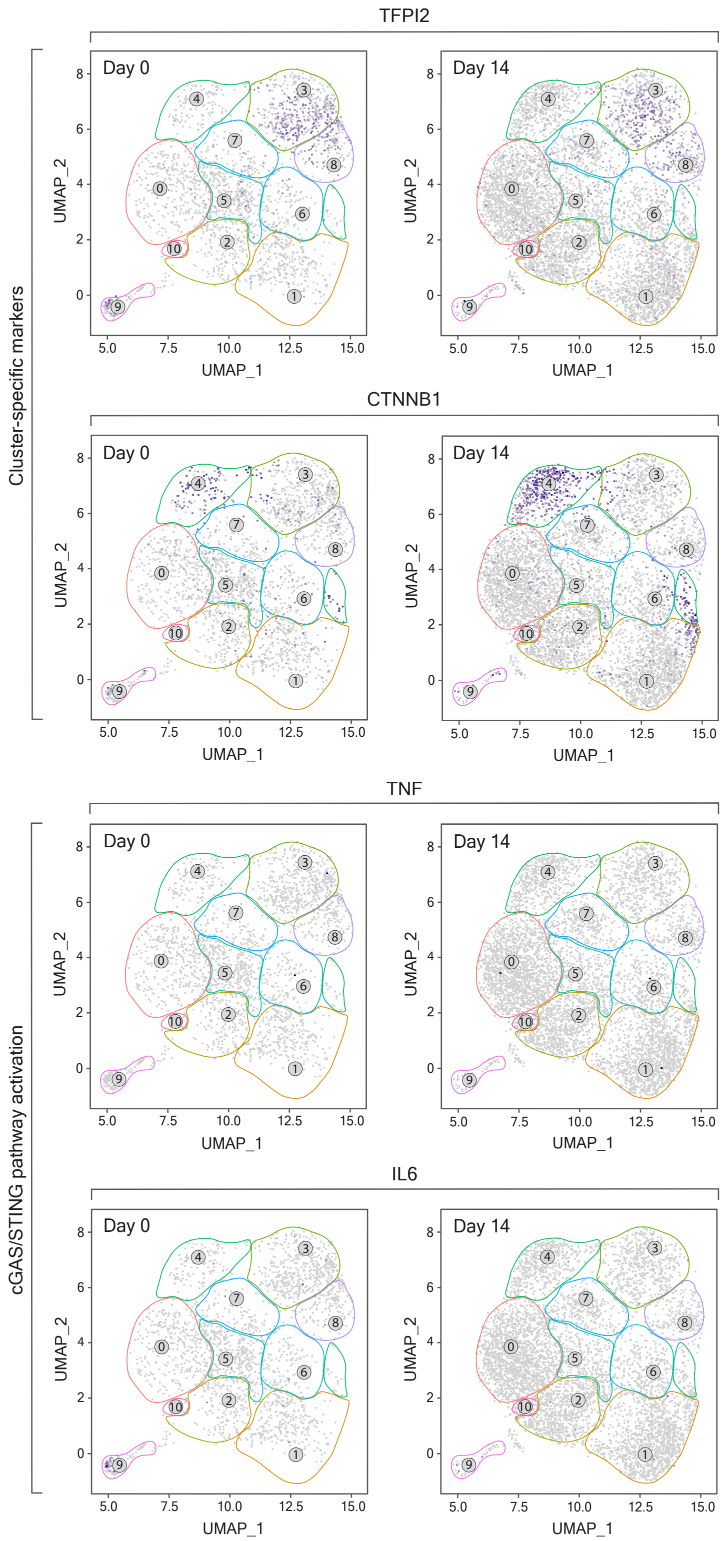

Supplementary Figure 4. BdLT-Seq episomes do not engage the exogenous DNA recognition machinery. UMAP plots of scRNA-Seq data obtained from HA1ER F12 cells (Supplementary Fig. 2a) depicting the expression of TNF and IL6 mRNAs as surrogate markers for cGAS/STING pathway activation and TFPI2 and CTNNB1 mRNAs as examples of cluster specific transcriptome state marker.

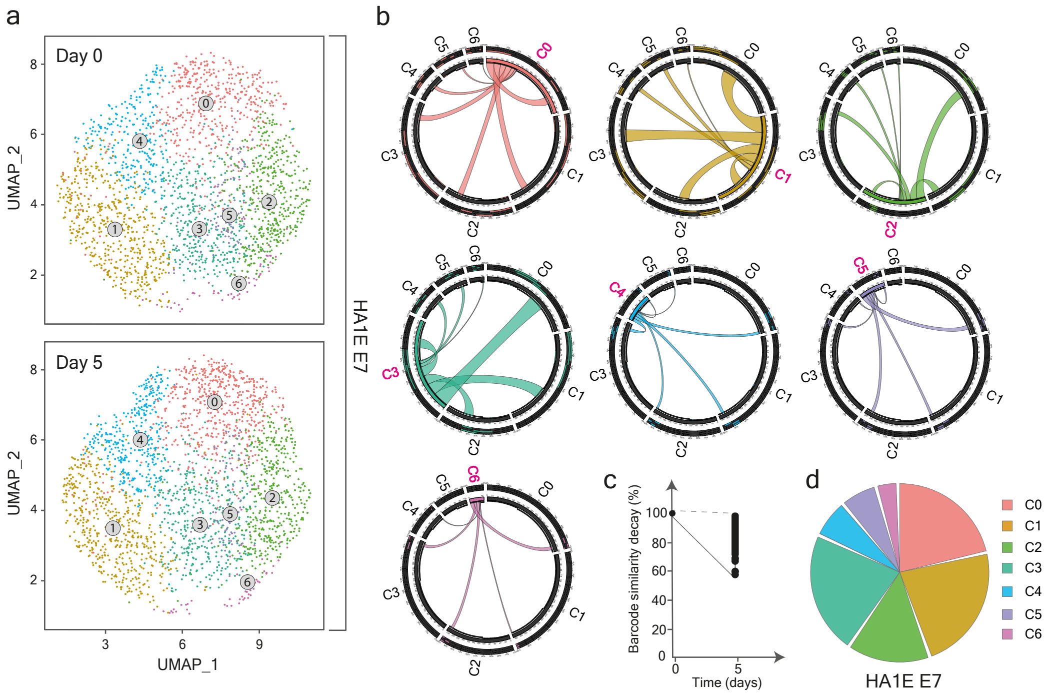

Supplementary Figure 5. BdLT-Seq unravels transcriptome plasticity in immortalized clonal HA1E cells. a- UMAP plots of scRNA-Seq data obtained from HA1E E7 cells and traced using BdLT-Seq. Data obtained for Day 0 and Day 5 of tracing are shown. **b-** Chord diagrams representing transcriptome state dynamics for HA1E cells. All detected clusters are depicted (C0 to C6) and integrate the collapsed behaviour of all cells that belong to a particular gene expression state. Origin cluster is shown in pink (Day 0) and chords represent end point cluster association (Day 5). **c-** Scatter plot displays barcode similarity decay for all HA1E cells analysed after 5 days of tracing. d- Pie charts depicting the distribution of cells traced by BdLT-Seq for HA1E E7 matching Supplementary Fig. 5b. Source data are provided as a Source Data file.

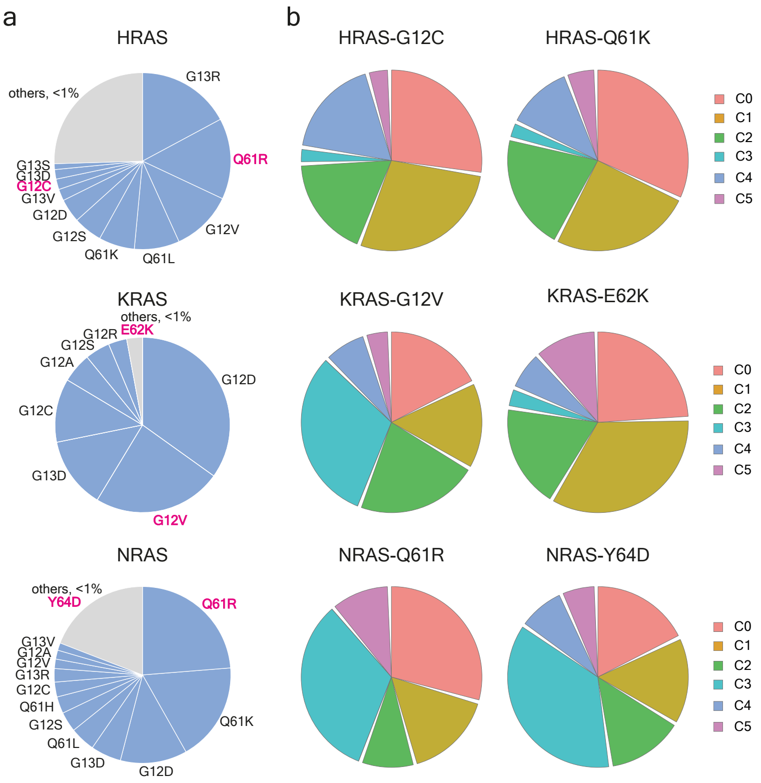

Supplementary Figure 6. scRNA-Seq revealed RAS variant-specific transcriptome states. a- Pie charts representing the pan-cancer frequency of individual amino acid substitutions for each RAS family, compiled from the Catalogue Of Somatic Mutations In Cancer (COSMIC v9248). The most common RAS mutations accounting for >1% of all cancer-associated mutations in HRAS, KRAS or NRAS are indicated. The six mutant RAS variants included in the multiplexed BdLT-Seq experiment are highlighted in pink. **b-** Pie charts displaying the distribution of cells traced by BdLT-Seq for HRAS-G12C, HRAS-Q61R, KRAS-G12V, KRAS-E62K, NRAS-Q61R and NRAS-Y64D matching Fig. 2d. Source data are provided as a Source Data file.

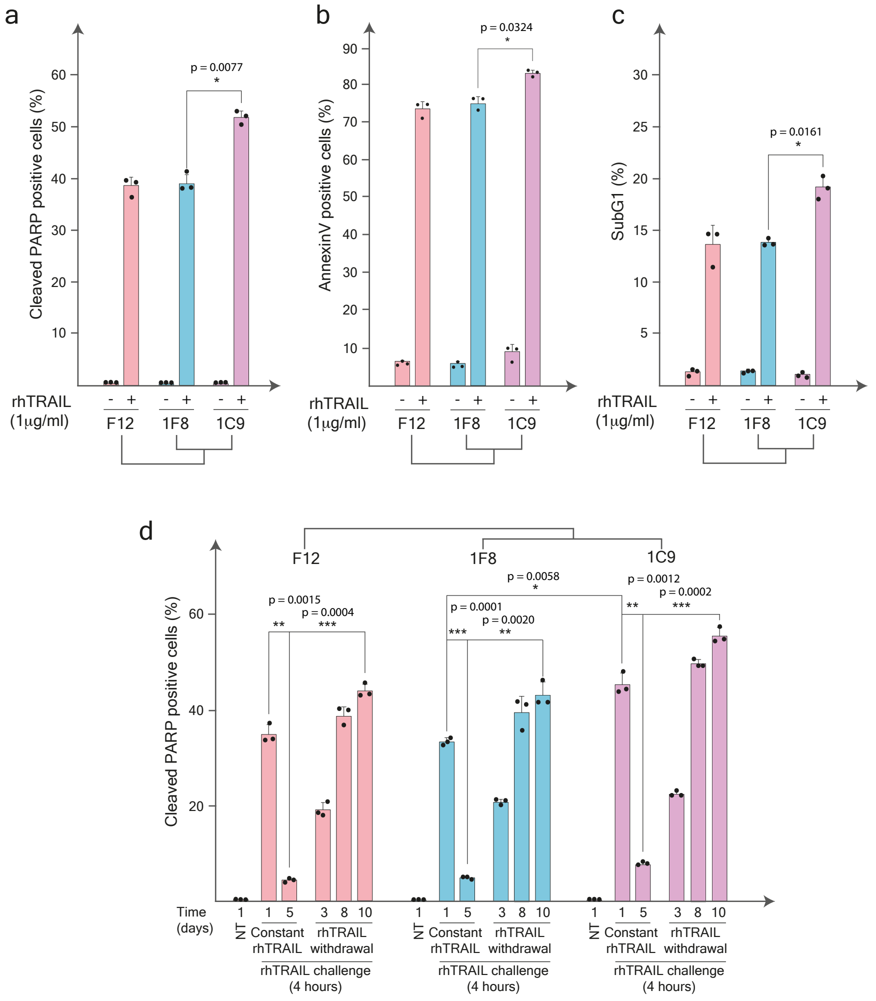

Supplementary Figure 7. TRAIL-induced transient resistance in clonal populations of HA1ER cells. a- Histogram displays the percentage of Cleaved PARP positive cells in control (-) and TRAIL treated (+) (4 hours, 1μg/ml) populations of HA1ER F12, 1F8 and 1C9 as analysed by flow cytometry. Histograms show mean value +/- standard deviation (SD) of three independent biological replicates (~20,000 cells per condition). Statistical significance was assessed by two-tailed paired Student's t-test. ***P-value < 0.0005, **P-value < 0.005, *P-value < 0.05. Clonal relationship is also depicted. Individual data points are shown. Displayed histogram is a subset from a data set shown in Fig. 3b. **b-** Histogram displays the percentage of Annexin V positive cells in control (-) and TRAIL treated (+) (4 hours, 1μg/ml) populations of HA1ER F12, 1F8 and 1C9 as analysed by flow cytometry. Histograms show mean value +/- standard deviation (SD) of three independent biological replicates (~20,000 cells per condition). Statistical significance was assessed by two-tailed paired Student's t-test. ***P-value < 0.0005, **P-value < 0.005, *P-value < 0.05. Clonal relationship is also depicted. Individual data points are shown. **c-** Histogram shows the percentage of cells in SubG1 in control (-) and TRAIL treated (+) (16 hours, 1μg/ml) populations of HA1ER F12, 1F8 and 1C9 cells as analysed by DAPI staining using flow cytometry. Histograms show mean value +/- standard deviation (SD) of three independent biological replicates (~20,000 cells per condition). Statistical significance was assessed by two-tailed paired Student's t-test. ***P-value < 0.0005, **P-value < 0.005, *P-value < 0.05. Clonal relationship is also depicted. Individual data points are shown. **d-** Histogram depicting TRAIL-induced apoptosis in HA1ER parental clone (F12, pink) and two F12-subclones (1F8 and 1C9, blue and light purple respectively) as determined by cleaved PARP staining analysed by flow cytometry. No TRAIL (NT) and constant rhTRAIL treatment are depicted as controls of acquired TRAIL resistance. Day 3, Day 8 and Day 10 of rhTRAIL withdrawal is shown to denote escape from TRAIL resistance and reversion to fractional killing induced by TRAIL. Histograms show mean value +/- standard deviation (SD) of three independent biological replicates (~20,000 cells per condition). Statistical significance was assessed by two-tailed paired Student's t-test. ***P-value < 0.0005, **P-value < 0.005, *P-value <0.05. Clonal relationship is also depicted. Individual data points are shown. Source data are provided as a Source Data file.

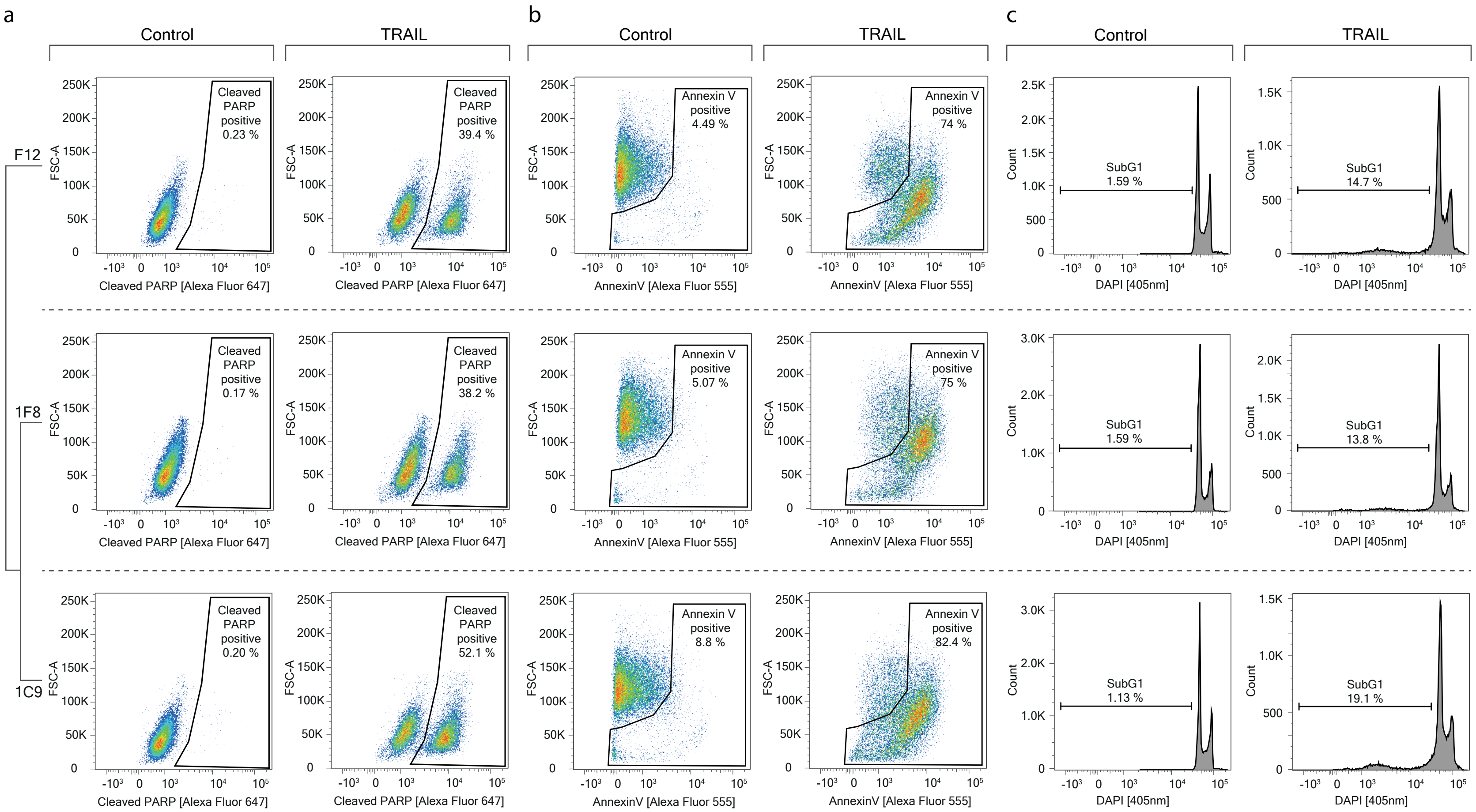

Supplementary Figure 8. Gating strategy for TRAIL-induced apoptosis measured by Flow Cytometry. a- Scatter plots displaying the percentage of cleaved PARP positive cells in control and TRAIL treated (4 hours, 1μg/ml) populations (Control, TRAIL) of HA1ER F12, 1F8 and 1C9 as analysed by flow cytometry. Images from one representative experiment are shown. Percentage of cleaved PARP positive cells is indicated. **b-** Scatter plots showing the percentage of Annexin V positive cells in control and TRAIL treated (4 hours, 1μg/ml) populations (Control, TRAIL) of HA1ER F12, 1F8 and 1C9 as analysed by flow cytometry. Images from one representative experiment are shown. Percentage of Annexin V positive cells is indicated. **c-** Histograms show the percentage of cells in SubG1 in control and TRAIL treated (16 hours, 1μg/ml) populations (Control, TRAIL) of HA1ER F12, 1F8 and 1C9 cells as analysed by DAPI staining using flow cytometry. Images from one representative experiment are shown. Percentage of cells in SubG1 is indicated.

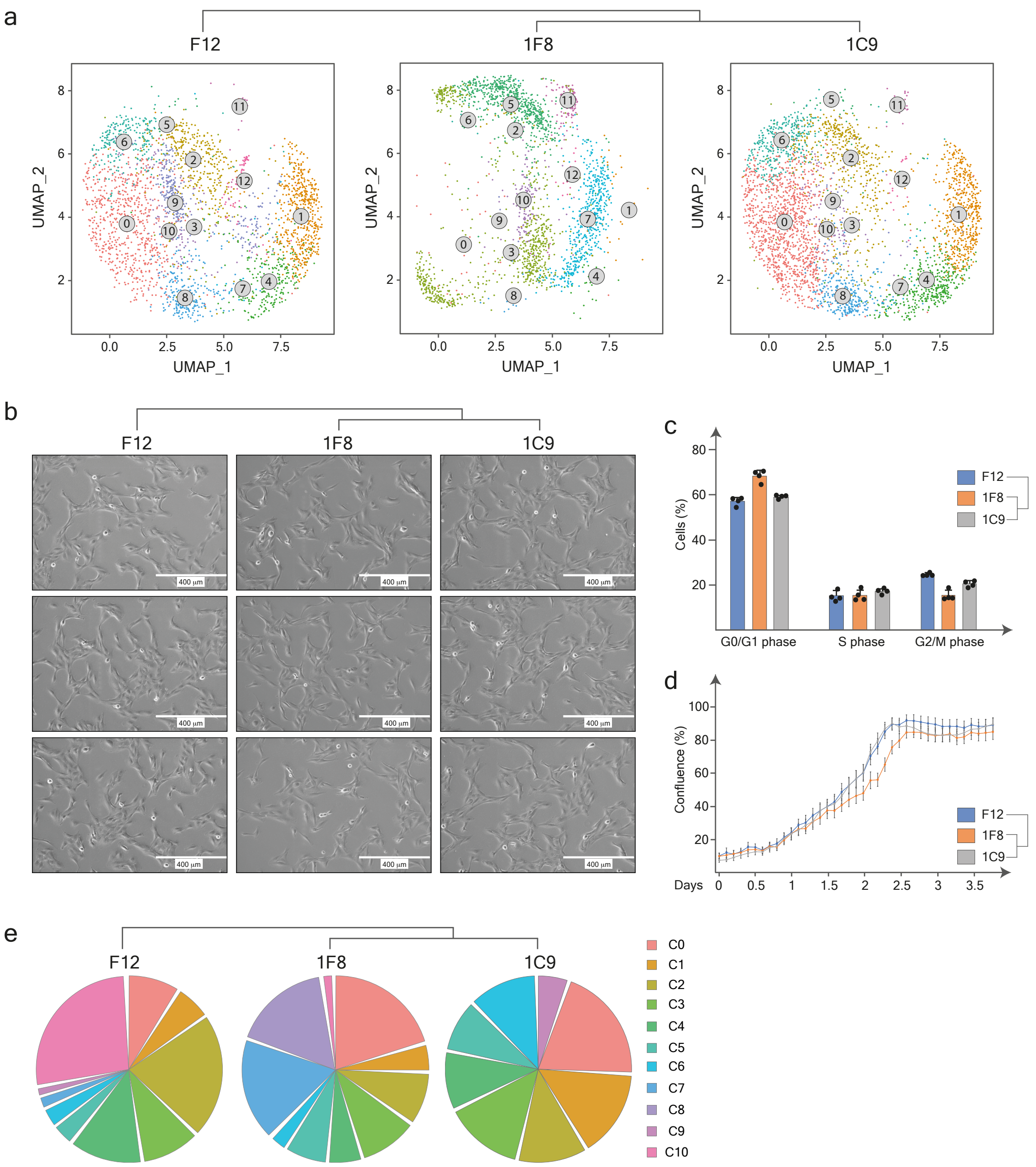

Supplementary Figure 9. Transcriptome divergence in sub-clonal populations of HA1ER cells. a- UMAP plots of scRNA-Seq data obtained from HA1ER F12 clone and its subclones 1C9 and 1F8. **b-** Micrographs depicting HA1ER F12, 1F8 and 1C9 cells. Scale bar represents 400µm. **c-** Histogram shows the representation of cells in each cell cycle phase for HA1ER F12, 1F8 and 1C9 cells as analysed by DAPI staining using flow cytometry. Histograms show mean value +/- standard deviation (SD) of at least three independent biological replicates. Individual data points are shown. **d-** Line plot represents proliferation curves for HA1ER F12, 1F8 and 1C9 cells as determined by IncuCyte imager. Results presented in the graph were obtained from at least two independent biological replicates imaging 36 fields per replicate. **e-** Pie charts displaying the transcriptome state distribution of HA1ER F12, HA1ER 1F8, HA1ER 1C9 cells traced by BdLT-Seq matching Fig. 3d. Source data are provided as a Source Data file.

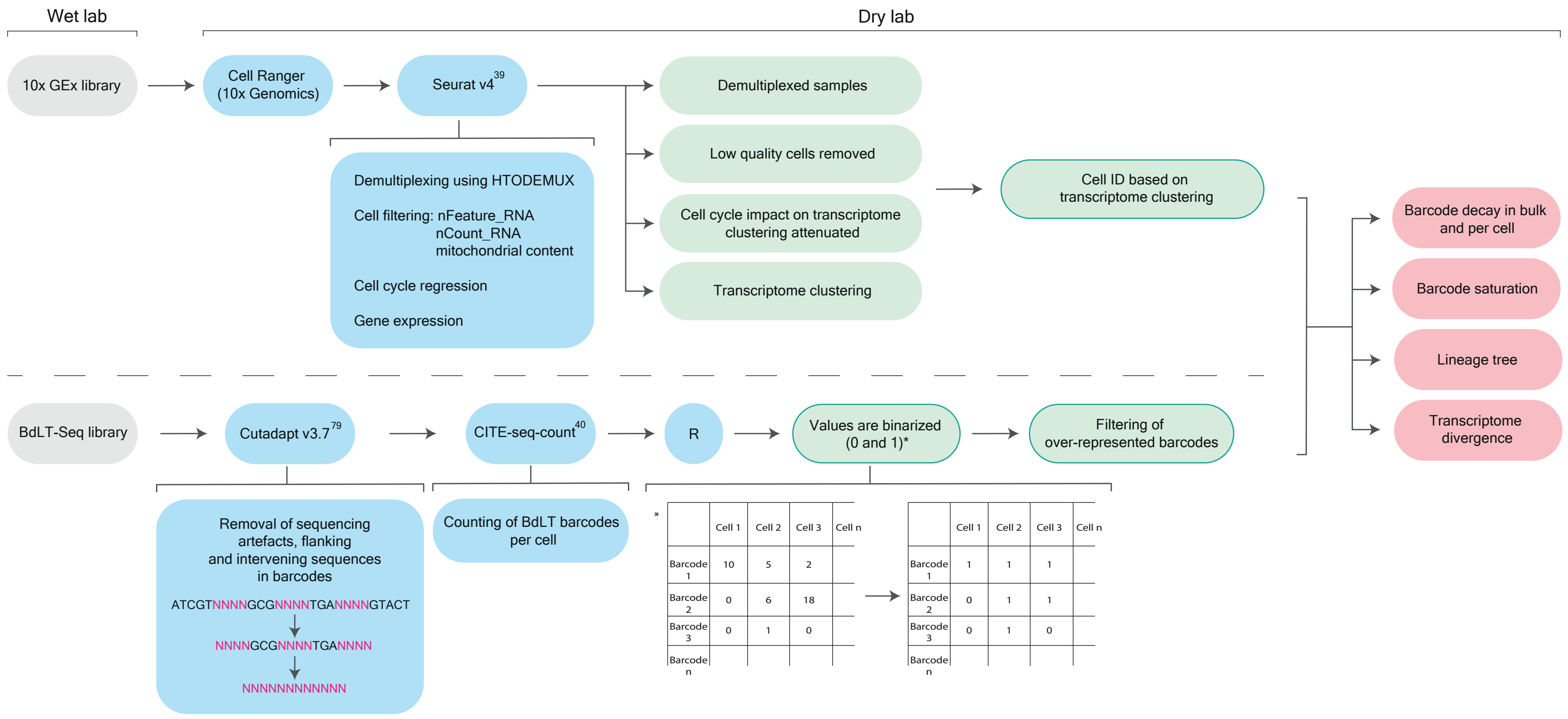

Supplementary Figure 10. Computational pipeline for BdLT-Seq data analysis. Scheme representing computational pipeline used for BdLT-Seq analysis. Two independent libraries - one for gene expression (10x GEx library) and the other one for lineage tracing (BdLT-Seq library) are prepared. Independent computational pipelines are applied for data analysis and combined as depicted.
