# Supplementary material for: BdLT-Seq as a barcode decay-based method to unravel lineage-linked transcriptome plasticity
Source: Nat Commun. 2023 Feb 25;14:1085. doi: 10.1038/s41467-023-36744-1 (PMC9968323; doi:10.1038/s41467-023-36744-1)
Supplement: Supplementary file 2 — Description of Additional Supplementary Files [file 41467_2023_36744_MOESM2_ESM.pdf]

Description of Additional Supplementary Files

Supplementary Data 1. List of primers used for BdLT-Seq. Table specifying primer sequences used to generate LTv-BC-H2B-GFP/mCherry episome libraries, barcoded RAS-encoding lentiviral vector, and sequencing libraries. The list also includes sequences of specific probes used for LTv-BC-H2B-GFP and LTv-BC-mCherry capturing, barcodes associated with specific RAS variants and qPCR primers used for LTv-BC-H2B-GFP RT-qPCR. Lineage tracing barcodes and indexes in primers used to generate sequencing libraries are highlighted in red.

Supplementary Code. Node script to determine barcode similarity between data points used to reconstruct lineage relationships.
