# Supplementary material for: BdLT-Seq as a barcode decay-based method to unravel lineage-linked transcriptome plasticity
Source: Nat Commun. 2023 Feb 25;14:1085. doi: 10.1038/s41467-023-36744-1 (PMC9968323; doi:10.1038/s41467-023-36744-1)
Supplement: Supplementary file 5 — Reporting Summary [file 41467_2023_36744_MOESM5_ESM.pdf]

nature portfolio

Corresponding author(s): Maximiliano Portal

Last updated by author(s): 27/01/2023

# Reporting Summary

Nature Portfolio wishes to improve the reproducibility of the work that we publish. This form provides structure for consistency and transparency in reporting. For further information on Nature Portfolio policies, see our Editorial Policies and the Editorial Policy Checklist.

## Statistics

For all statistical analyses, confirm that the following items are present in the figure legend, table legend, main text, or Methods section.

| n/a | Confirmed | |
|---|---|---|
| ☐ | ☒ | The exact sample size (*n*) for each experimental group/condition, given as a discrete number and unit of measurement |
| ☐ | ☒ | A statement on whether measurements were taken from distinct samples or whether the same sample was measured repeatedly |
| ☐ | ☒ | The statistical test(s) used AND whether they are one- or two-sided<br>*Only common tests should be described solely by name; describe more complex techniques in the Methods section.* |
| ☒ | ☐ | A description of all covariates tested |
| ☒ | ☐ | A description of any assumptions or corrections, such as tests of normality and adjustment for multiple comparisons |
| ☐ | ☒ | A full description of the statistical parameters including central tendency (e.g. means) or other basic estimates (e.g. regression coefficient) AND variation (e.g. standard deviation) or associated estimates of uncertainty (e.g. confidence intervals) |
| ☐ | ☒ | For null hypothesis testing, the test statistic (e.g. *F*, *t*, *r*) with confidence intervals, effect sizes, degrees of freedom and *P* value noted<br>*Give P values as exact values whenever suitable.* |
| ☒ | ☐ | For Bayesian analysis, information on the choice of priors and Markov chain Monte Carlo settings |
| ☒ | ☐ | For hierarchical and complex designs, identification of the appropriate level for tests and full reporting of outcomes |
| ☒ | ☐ | Estimates of effect sizes (e.g. Cohen's *d*, Pearson's *r*), indicating how they were calculated |

*Our web collection on statistics for biologists contains articles on many of the points above.*

## Software and code

Policy information about availability of computer code

| | |
|---|---|
| Data collection | - FACS data - BD FACSAria III Cell Sorter (BD Biosciences), BD InFlux Cell Sorter (BD Biosciences), BD LSRFortessa X-20 Cell Analyzer, BD Biosciences.<br>- IncuCyte data - IncuCyte S3 Life Cell Analysis Instrument (Sartorius, cat. #4647).<br>- scRNA-Seq - NovaSeq 6000 version 1.5 (Illumina, cat. #20013850).<br>- Barcode count - NovaSeq 6000 version 1.5 (Illumina, cat. #20013850)<br>- qPCR - QuantStudio3 Real Time PCR System (Thermo Fisher Scientific, cat. #A28136).<br>- Micrographs - inverted microscope (ThermoFisher Scientific, cat. #AMEX1000). |
| Data analysis | - FACS data - FlowJo software (BD, version 10.8.0).<br>- IncuCyte data - IncuCyte version 2020C Rev1 software.<br>- scRNA-Seq data - Seurat v4.<br>- BdLT-Seq data - CITE-seq-count, custom UNIX and R scripts and cutadapt v3.7.<br>- qPCR - Real Time qPCR Connect Data Analysis Tool (Thermo Fisher Scientific Connect Platform).<br>- R version 4.2.0.<br>- CIRCOS version 0.69-9.<br>- pan-cancer frequency of individual amino acid substitutions for each RAS family (Catalogue Of Somatic Mutations In Cancer (COSMIC v92)). |

For manuscripts utilizing custom algorithms or software that are central to the research but not yet described in published literature, software must be made available to editors and reviewers. We strongly encourage code deposition in a community repository (e.g. GitHub). See the Nature Portfolio guidelines for submitting code & software for further information.

## Data

Policy information about availability of data

All manuscripts must include a data availability statement. This statement should provide the following information, where applicable:

- Accession codes, unique identifiers, or web links for publicly available datasets
- A description of any restrictions on data availability
- For clinical datasets or third party data, please ensure that the statement adheres to our policy

Sequencing data is deposited to the Gene Expression Omnibus database under the accession code GSE223496 (https://www.ncbi.nlm.nih.gov/geo/query/acc.cgi?acc=GSE223496). Source data are provided with this paper. The raw data is provided for the following figures: Figure 1c-i; Figure 2b-d; Figure 3b-d; Supplementary Figure 1 e-g; Supplementary Figure 2a-c and e; Supplementary Figure 3a; Supplementary Figure 4a; Supplementary Figure 5a-d; Supplementary Figure 6a-b, Supplementary Figure 7 a-d, Supplementary Figure 9 a-e.

## Human research participants

Policy information about studies involving human research participants and Sex and Gender in Research.

| | |
|---|---|
| Reporting on sex and gender | *Use the terms sex (biological attribute) and gender (shaped by social and cultural circumstances) carefully in order to avoid confusing both terms. Indicate if findings apply to only one sex or gender; describe whether sex and gender were considered in study design whether sex and/or gender was determined based on self-reporting or assigned and methods used. Provide in the source data disaggregated sex and gender data where this information has been collected, and consent has been obtained for sharing of individual-level data; provide overall numbers in this Reporting Summary. Please state if this information has not been collected. Report sex- and gender-based analyses where performed, justify reasons for lack of sex- and gender-based analysis.* |
| Population characteristics | *Describe the covariate-relevant population characteristics of the human research participants (e.g. age, genotypic information, past and current diagnosis and treatment categories). If you filled out the behavioural & social sciences study design questions and have nothing to add here, write "See above."* |
| Recruitment | *Describe how participants were recruited. Outline any potential self-selection bias or other biases that may be present and how these are likely to impact results.* |
| Ethics oversight | *Identify the organization(s) that approved the study protocol.* |

Note that full information on the approval of the study protocol must also be provided in the manuscript.

# Field-specific reporting

Please select the one below that is the best fit for your research. If you are not sure, read the appropriate sections before making your selection.

[x] Life sciences [ ] Behavioural & social sciences [ ] Ecological, evolutionary & environmental sciences

For a reference copy of the document with all sections, see nature.com/documents/nr-reporting-summary-flat.pdf

# Life sciences study design

All studies must disclose on these points even when the disclosure is negative.

| | |
|---|---|
| Sample size | - All histograms displayed in the manuscript show mean value +/- standard deviation (SD) of two-three independent biological replicates (~20,000 - 30,000 cells per condition). The sample size was determined by assessing the number of singlets using BD LSRFortessa X-20 Cell Analyzer and FlowJo software (BD, version 10.8.0).<br>- scRNA-Seq - 10,000 cells were processed per experiment. After data per-processing, approximately 7,000 - 8,000 cells were taken forward for data analysis. The sample size was determined by assessing the number of unique cell identifiers using Seurat v4. |
| Data exclusions | As part of scRNA-Seq data pre-processing we mask cells displaying high levels of mitochondrial transcripts, abnormal read counts and perform cell cycle regression which can result in further drop-outs. We also exclude cells displaying multiple HTO labeling after demultiplexing. |
| Replication | - All histograms displayed in the manuscript show mean value +/- standard deviation (SD) of two- three independent biological replicates (~20,000 - 30,000 cells per condition). Statistical significance was assessed by two-tailed paired Student's t-test. ***P-value < 0.0005, **P-value < 0.005. Individual data measurements are depicted.<br>- IncuCyte data - Results presented in the graph were obtained from two independent biological replicates imaging 36 fields per replicate.<br>- Micrographs - Representative images (out of at least 5 biological replicates) are shown.<br>All attempts of replication were successful. Each experiment was performed at least two - three times (independent biological replicates). |
| Randomization | No sample randomization was used in any of our experiments. Random sampling was only applied to BdLT-Seq data analysis to reduce the number of barcodes used to build lineage trees (Figure ld) to reduce computational burden. |

| | |
|---|---|
| Blinding | Not relevant to our study due to the time-resolved nature of the samples analyzed in our manuscript. The relationship between time-resolved samples must be known for data analysis and interpretation. |

# Reporting for specific materials, systems and methods

We require information from authors about some types of materials, experimental systems and methods used in many studies. Here, indicate whether each material, system or method listed is relevant to your study. If you are not sure if a list item applies to your research, read the appropriate section before selecting a response.

## Materials & experimental systems

| n/a | Involved in the study | |
|---|---|---|
| ☐ | ☒ | Antibodies |
| ☐ | ☒ | Eukaryotic cell lines |
| ☒ | ☐ | Palaeontology and archaeology |
| ☒ | ☐ | Animals and other organisms |
| ☒ | ☐ | Clinical data |
| ☒ | ☐ | Dual use research of concern |

## Methods

| n/a | Involved in the study | |
|---|---|---|
| ☒ | ☐ | ChIP-seq |
| ☐ | ☒ | Flow cytometry |
| ☒ | ☐ | MRI-based neuroimaging |

# Antibodies

| | |
|---|---|
| Antibodies used | - Cleaved PARP (Asp214) (D64E10) XP Rabbit mAb (Alexa Fluor 647 Conjugate) (Cell Signalling, cat. #6987).<br>- Annexin V (Invitrogen, cat. # A35108).<br>- Hashtag antibodies (BioLegend, cat. #A0251, clone LNH-94 2M2, Lot B342028; A0252, clone LNH-94 2M2, Lot B363811; A0253, clone LNH-94 2M2, Lot B315096; A0254, clone LNH-94 2M2, Lot B317177; A0255, clone LNH-94 2M2, Lot B366838; A0256, clone LNH-94 2M2, Lot B341500; A0257, clone LNH-94 2M2, Lot B272606; A0258, clone LNH-94 2M2, Lot B272605; A0259, clone LNH-94 2M2, Lot B277212; A0260, clone LNH-94 2M2, Lot B277179). |
| Validation | - Cleaved PARP antibodies were validated by assessing TRAIL-induced apoptosis in HA1ER cells (Figure 3b, Supplementary Figure 7a and d).<br>- Annexin V antibodies were validated by assessing TRAIL-induced apoptosis in HA1ER cells (Supplementary Figure 7b).<br>- Hashtag antibodies were validated by successful demultiplexing of multiple samples in scRNA-Seq data sets (Figure 1e-g, Figure 2b, Figure 3c, Supplementary Figure 2a, Supplementary Figure 4a, Supplementary Figure 5a, Supplementary Figure 9a). |

# Eukaryotic cell lines

Policy information about cell lines and Sex and Gender in Research

| | |
|---|---|
| Cell line source(s) | - Immortalised HA1E (hTERT and SV40 ER) and tumorigenic HA1ER cells (hTERT, SV40ER and HRAS-G12V) from stepwise tumorigenesis models generated from normal human embryonic kidney cells were obtained from Dr. Hahn (Broad Institute of MIT and Harvard, Cambridge, USA).<br>- LentiX 293T (Takara, cat. #632180) - a subclone of the transformed human embryonic kidney cell line, HEK293, were obtained from Prof. Lacaud (Cancer Research UK Manchester Institute, Manchester, UK). |
| Authentication | Cell line authentication was performed using Verogen MainstAY Kit for Short Tandem Repeat profiling which contains 27 autosomal STRs and 25 Y-STR markers. |
| Mycoplasma contamination | We confirm that all cell lines tested negative for mycoplasma contamination. |
| Commonly misidentified lines<br>(See ICLAC register) | No commonly misidentified cell lines were used in the study. |

# Flow Cytometry

## Plots

Confirm that:

☒ The axis labels state the marker and fluorochrome used (e.g. CD4-FITC).

☒ The axis scales are clearly visible. Include numbers along axes only for bottom left plot of group (a 'group' is an analysis of identical markers).

☐ All plots are contour plots with outliers or pseudocolor plots.

☒ A numerical value for number of cells or percentage (with statistics) is provided.

## Methodology

| | |
|---|---|
| Sample preparation | - To determine the percentage of cleaved-PARP positive cells, cells were fixed with ice-cold methanol. After methanol fixation, cells were washed once with 1x PBS followed by blocking with 0.5% BSA PBS for 1hour at 20°C. Immunolabelling of cleaved-PARP was performed using fluorescently labeled cleaved-PARP Alexa 647 antibody (Cell Signaling, cat. #6987) for 1 hour at 20°C. Next, cells were washed once with PBS followed by flow cytometry analysis using BD LSRFortessa X-20 Cell Analyzer (BD Biosciences). Approximately 20,000 cells were analyzed for each biological replicate.<br>- To determine the percentage of Annexin V positive cells, cells were trypsinised and collected, washed once with 1x PBS followed by labeling with Annexin V antibody in annexin-binding buffer (10 mM HEPES, 140 mM NaCl, and 2.5 mM CaCl2, pH 7.4) for 15 min at room temperature. Data acquisition and analysis was performed using BD LSRFortessa X-20 Cell Analyzer (BD Biosciences). Approximately 20,000 cells were analyzed for each biological replicate.<br>- For cell cycle and SubG1 analysis, cells were collected, fixed and permeabilised with ice-cold methanol. After methanol fixation, cells were washed once with PBS followed by blocking with 0.5% BSA PBS for 1hour at 20°C. DAPI labeling was performed for 30min at 20°C. Next, cells were washed once with PBS followed by flow cytometry analysis using BD LSRFortessa X-20 Cell Analyzer (BD Biosciences). Approximately 30,000 cells were analyzed for each sample.<br>- For live cell analysis or sorting of GFP or mCherry positive cells, cells were collected by trypsinisation, centrifuged, resuspended in medium and GFP or mCherry positive cells were sorted using BD FACSAria III Cell Sorter (BD Biosciences) or BD InFlux Cell Sorter (BD Biosciences). |
| Instrument | BD FACSAria III Cell Sorter (BD Biosciences), BD InFlux Cell Sorter (BD Biosciences), BD LSRFortessa X-20 Cell Analyzer (BD Biosciences). |
| Software | FlowJo software (BD, version 10.8.0). |
| Cell population abundance | The purity check of GFP or mCherry positive populations was performed after every sort, by acquiring approximately 1,000 cells from sorted samples. The abundance of the relevant populations in the sorted fractions varied from 94% to 99%. |
| Gating strategy | Initial FSC vs SSC gating was used to identify the cells of interest based on their size and granularity, excluding cell debris. Next FSC-H vs FSC-A plots was used to exclude doublets. Single parameter density plots or histograms were used to identify the cells expressing a marker of interest (GFP, mCherry, cleaved PARP, Annexin). Two parameter density plots were used to determine the cells expressing both markers of interest (GFP and mCherry). |

☒ Tick this box to confirm that a figure exemplifying the gating strategy is provided in the Supplementary Information.
